# Multivariate inference of pathway activity in host immunity and response to therapeutics

**DOI:** 10.1093/nar/gku722

**Published:** 2014-08-21

**Authors:** Gautam Goel, Kara L. Conway, Martin Jaeger, Mihai G. Netea, Ramnik J. Xavier

**Affiliations:** 1 Center for Computational and Integrative Biology, Massachusetts General Hospital, Boston, MA 02114, USA; 2 Gastrointestinal Unit and Center for the Study of Inflammatory Bowel Disease, Massachusetts General Hospital and Harvard Medical School, Boston, MA 02114, USA; 3 Broad Institute of Harvard University and Massachusetts Institute of Technology, Cambridge, MA 02142, USA; 4 Department of Internal Medicine and Nijmegen Institute for Infection, Inflammation and Immunity, Radboud University Nijmegen Medical Centre, Nijmegen 6525 GA, The Netherlands

## Abstract

Developing a quantitative view of how biological pathways are regulated in response to environmental factors is central for understanding of disease phenotypes. We present a computational framework, named Multivariate Inference of Pathway Activity (MIPA), which quantifies degree of activity induced in a biological pathway by computing five distinct measures from transcriptomic profiles of its member genes. Statistical significance of inferred activity is examined using multiple independent self-contained tests followed by a competitive analysis. The method incorporates a new algorithm to identify a subset of genes that may regulate the extent of activity induced in a pathway. We present an in-depth evaluation of specificity, robustness, and reproducibility of our method. We benchmarked MIPA's false positive rate at less than 1%. Using transcriptomic profiles representing distinct physiological and disease states, we illustrate applicability of our method in (i) identifying gene–gene interactions in autophagy-dependent response to *Salmonella* infection, (ii) uncovering gene–environment interactions in host response to bacterial and viral pathogens and (iii) identifying driver genes and processes that contribute to wound healing and response to anti-TNFα therapy. We provide relevant experimental validation that corroborates the accuracy and advantage of our method.

## INTRODUCTION

Genome-wide association studies (GWAS) and next-generation sequencing technologies are rapidly providing insights into genetic definitions of host susceptibility to complex diseases (1–3). However, these approaches bring with them the formidable task of deriving a functional understanding of how genetic variants lead to the dysregulation of molecular pathways that underlies disease. The bottleneck here is technologies that can directly report on the functional state of a multitude of pathways that are simultaneously active or dysregulated in disease. Expression microarray or RNA-seq technologies enable comprehensive monitoring of expression levels of genes but pose the additional challenge of convolving such data into quantitative pathway-centric measures. Conceptually, such a computationally inferred metric would be reflective of the functional ‘activity’ induced in a pathway and correlated to a macroscopic property such as metabolic flux or signal flow. Identification of robust pathway activity measures continues to be an active area of research for functional class scoring (FCS) methods and recent studies have demonstrated their utility in analysis of expression data from single perturbation studies or case-control studies (4–13).

FCS methods typically aggregate a gene-level statistic into a single pathway-level metric, which could be either univariate, such as mean or median of expression levels (5), or multivariate, such as the Hotelling's *T*^2^ statistic (14). Comparative analyses have demonstrated considerable differences in performance between such measures. Hwang *et al.* (15) showed that these methods tend to produce discordant pathway activity signatures between related but independent datasets. Glazko *et al.* (16) showed that irrespective of the size and proportion of differentially expressed genes, the power of any aggregate statistic is inversely proportional to the amount of correlation present between genes. It was hoped that pathway-based methods would be an effective means of marker identification and disease classification, but typically they have not been able to outperform gene-based methods.

Here, we present a computational method, named Multivariate Inference of Pathway Activity (MIPA), which employs a principal component analysis (PCA)-based clustering framework and uses five distinct clustering-based summary statistics to assess the magnitude and significance of induced activity. A competitive analysis ensures that the observed activity level is more significant than that estimated for a random gene set with a comparable number of genes. The method incorporates a new algorithm to identify a subset of genes that may regulate the extent of activity induced in a pathway. We demonstrate that our metric is linearly sensitive to changes in both magnitude and proportion of differentially expressed genes. MIPA's sensitivity to changes in correlation structure enables us to discriminate between instances when the flux through a pathway is conserved versus when the flux is re-routed. We present an in-depth evaluation of specificity, robustness and reproducibility of our method. We benchmarked MIPA's false positive rate at less than 1%. Using transcriptomic profiles representing distinct physiological and disease states, we illustrate applicability of our method in (i) identifying gene–gene interactions in autophagy-dependent response to *Salmonella* infection, (ii) uncovering gene-environment interactions in host response to bacterial and viral pathogens and (iii) identifying driver genes and processes that contribute to wound healing and response to anti-TNFα therapy. We provide relevant experimental validation that corroborates the accuracy and advantage of our method.

### Approach

**Figure 1.**
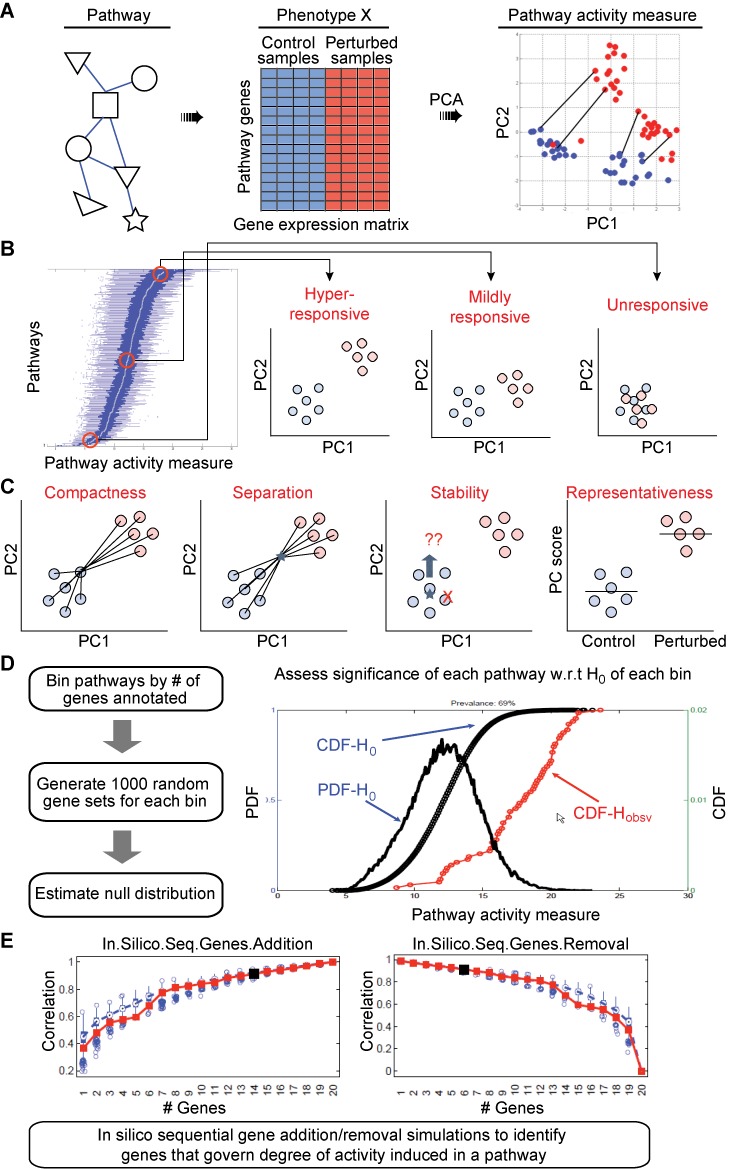
A framework for pathway activity inference. (**A**) A PCA-based clustering framework is used to assess the phenotypic separation between the basal and perturbed states. The degree of activity induced is quantified by computing the Euclidean distance between paired samples. (**B**) Based on the distribution of activity induced in samples from the perturbed group, the pathways are ranked as hyper-responsive, mildly responsive and unresponsive. (**C**) Four clustering-based summary statistics are computed to assess the magnitude and significance of induced activity. (**D**) Significance of observed activity is compared with that estimated for a random gene set with comparable number of genes. (**E**) Up to 1000 *in silico* simulations are executed in which genes are sequentially added or removed to identify the minimum set of genes that would recover 90% or more of the pathway activity level observed with the full set.

Our pathway activity inference method is based on the following two premises: first, it is assumed that if a pathway were transcriptionally regulated in a perturbed state, then that set of samples would cluster together and be distinguishable from the set of control samples. This phenotypic separation would exist in some feature dimension space determined by gene expression data. Second, the magnitude of separation between these clusters would be indicative of the degree of activity induced in that pathway (Figure 1A).

In the present iteration, a PCA-based clustering framework is used to assess the phenotypic separation between the basal and perturbed states. The degree of activity induced is quantified by computing the Euclidean distance between paired samples in the *n*-dimensional principal components space, where *n* is determined by the number of principal components required to explain at least 90% of the variance in the data. In the case of non-paired samples, Mahalanobis distance is computed between each perturbed sample and cluster of control samples. Based on the distribution of activity induced in samples from the perturbed group, the pathways are ranked as hyper-responsive, mildly responsive or unresponsive (Figure 1B).

Besides the primary pathway activity measure, four additional parameters are computed to evaluate the clustering tendency of two phenotypically distinct groups. Any given pathway, if truly activated, would maximize these parameters (Figure 1C): (1) *compactness*, which ensures that intra-cluster variability is small relative to the average inter-cluster variability, (2) *separation*, which ensures maximum separation between centroids of two clusters, (3) *stability*, which checks whether the clusters are overly sensitive to each sample within a group, and (4) *representativeness*, which ensures that a pathway consists of genes that are representative of a particular phenotype. The exact computations are outlined in the ‘Materials and Methods’ section.

We implemented a three-stage analytical pipeline to perform the following computations: beginning with an expression matrix of genes annotated in a pathway, Stage 1 (Figure 1C) computes five clustering-based measures and assesses their significance using independent self-contained tests; Stage 2 (Figure 1D) estimates the competitive significance of each pathway; and Stage 3 (Figure 1E) identifies a subset of *driver* genes that govern the degree of activity induced in each significant pathway. In Stage 1, sample labels are randomly permuted to generate null distributions for each parameter and significance of the estimated parameter is evaluated against these distributions. This is followed by a competitive analysis in Stage 2 to ensure that the observed activity level assessed with the given set of gene annotations is more significant than the activity level estimated for a random gene set with a comparable number of genes. *P*-values for the competitive test are estimated using a Student's *t*-test on distribution of pathway activity measure alone (distribution being derived from activity estimate per sample) between the observed groups in a given pathway and the random gene sets simulated to contain similar number of genes (gene permutations). All *P*-value estimates are adjusted for multiple hypothesis testing correction using false discovery rate (FDR) controlling procedures. *P*-values from self-contained tests are first combined using Stouffer's *Z*-score method into an overall single *P*-value. Only pathways that score significantly on both resulting *P*-values (combined *P*-value of self-contained tests and *P*-value from competitive test) are considered significant. In Stage 3, up to 1000 *in silico* simulations are executed in which genes are sequentially added or removed to identify the minimum set of genes that would recover 90% or more of the pathway activity level observed with the full set. This subset is called the *driver* gene set for a pathway. The complete algorithm is outlined in the ‘Materials and Methods’ section.

## MATERIALS AND METHODS

### Pathway activity measure

Degree of pathway activity induced per sample is measured using either the Euclidean distance between paired samples or the Mahalanobis distance metric for non-paired samples. Unlike the Euclidean distance measure, Mahalanobis distance takes into account the covariance of the control samples and is computed as

\begin{equation*} d(i) = \sqrt {\sum\limits_{j = 1}^N {\left( {Y(i)_j - \mu _{{jx}} } \right)\times{S}^{ - 1} \times \left( {Y(i)_j - \mu _{{jx}} } \right)} } \end{equation*}

where *μ*_*jx*_ and *S* are the mean and covariance of the principal-component scores for the control samples.

### Compactness

Compactness of a cluster is estimated by computing the average Silhouette value over all data items. The Silhouette value for an individual data item, which reflects the confidence in a particular cluster assignment, is computed as

\begin{equation*} S_{i} = \frac{{{\bf b}_{{i}} - {\bf a}_{{i}} }}{{\max ({\bf b}_{{i}} ,{\bf a}_{{i}} )}} \end{equation*}

where **a***_i_* denotes the average distance between *i* and all data items in the same cluster, and **b***_i_* denotes the average distance between *i* and all data items in the closest other cluster. The Silhouette width is limited to the interval [−1,1] and should be maximized to ensure that intra-cluster variability is smaller relative to the average inter-cluster variability.

### Separation (Distance between the clusters)

Wards distance between clusters *C*_*i*_ and *C_j_* is computed as the difference between the total within-cluster sum of squares for the two clusters separately, and the within-cluster sum of squares resulting from merging the two clusters in cluster *C_ij_*.

\begin{eqnarray*} &&D_w (C_{i} ,C_{j} ) = \\ &&\sqrt {\sum\limits_{k = 1}^N {\left( {\sum\limits_{x \in C_{{ij}} } {(x - r_{{ij}} )^2 - \left[ {\sum\limits_{x \in C_{i} } {(x - r_{i} )^2 } + \sum\limits_{x \in C_{j} } {(x - r_{j} )^2 } } \right]} } \right)} } \end{eqnarray*}

where *r_i_* is centroid of *C_i_*, *r_j_* is centroid of *C_j_* and *r*_*ij*_ is centroid of *C_ij_*. This metric is similar to group average and centroid distance but less susceptible to noise and outliers. The distance between two clusters should be maximized.

### Stability/Robustness

A stable cluster is one that is robust to the removal of a small number of samples from the dataset. Using a jackknife approach, each sample is removed one at a time, following by re-computation of the difference between the centroids of the new clusters (in PCA space) from the original cluster. This average distance between means is computed as

\begin{equation*} D_{\rm m} (C_{j} ) = \frac{1}{N}\sum\limits_{s = 1}^N {d\left( {X_C^{{S},{i}} ,X_C^{{S},{\rm 0}} } \right)} \end{equation*}

where ${\rm X}_{\rm C}^{{S},0}$ denotes the average principal-components score profile for samples across cluster *C*_*s*,0_ with full data of all samples and ${\rm X}_{C}^{{S},{i}}$ denotes the average principal-components score profile for samples across cluster *C*_*s*,*i*_ obtained by removing one sample at a time. *N* is the number of samples. The average distance between means should be minimized.

### Representativeness/Informativeness

This metric examines whether a pathway consists of genes that are collectively representative of a particular phenotype. For a pathway *i* and its corresponding principal-component scores in each PC-dimension *j =* 1, 2, …*, N*, for the two phenotypic groups *k =* 1, 2 (for control and perturbed states), the following fixed effects model is fitted using one-way ANOVA:

\begin{equation*} P_{{jk}}^{(i)} = \mu + \mu _{{jk}} + \varepsilon _{{jk}} \end{equation*}

where *μ* reflects the overall mean, *μ_jk_* represents the effect of phenotype group *k* on the samples' principal-component score and ε*_jk_* is the random normal residual error term. Under the null hypothesis *H0*: $\mu _1 = \mu _2$, the assumption is that the two phenotypic group means are equivalent, or in other words, that there are no changes associated between perturbed and control states. From this ANOVA model, the F-statistic is computed as the ratio of the mean-squares from the two groups. The F-statistic captures the strength of association observed in a pathway's principal-component score profile over the different phenotype groups. Large values of the F-statistic indicate a strong association whereas a small F-statistic suggests that the samples demonstrate minimal phenotype-specific score changes.

### *Driver* gene identification

For each pathway, pairwise Euclidean distance between all pairs of samples (control and treatment) is computed in the PCA space. This set is designated as the null model PD_0_. The following steps are executed for up to 1000 iterations, dividing the samples at each iteration using a jackknife approach with 10% hold-out: (i) generate a randomly ordered sequence of annotated genes; (ii) sequentially add (or remove) one gene at a time to the pathway annotation; (iii) compute pair-wise Euclidean distance between all pairs of samples in PCA space (PD_G_); (iv) estimate correlation between PD_0_ (with full annotation) and PD_G_; (v) compute difference in correlation yielded by having added the new gene; (vi) for first 100 iterations, rank order genes by area under ROC curve (AUROC) and max-pairwise-correlation between genes; (vii) for remainder iterations, rank order genes by AUROC and history of difference in correlation yielded by each gene. (viii) At the end of 1000 iterations, the following steps are performed using the full dataset: (1) rank order genes by AUROC and history of difference in correlation yielded by each gene across 1000 iterations; (2) obtain correlation gain profile by adding one gene at a time; (3) obtain correlation loss profile by removing one gene at a time; and (4) select set of ‘*driver’* genes which when added sequentially yield a correlation ≥0.9.

### Performance benchmarking between MIPA variants

Six variants of MIPA were derived for benchmarking by considering two distinct types of competitive tests and three different ways of combining individual *P*-values (Figure 5). In variants 1–3, null distributions of all 5 MIPA measures were estimated by random permutation of gene labels and *P*-value estimates were calculated for a gene set in each observed dataset with respective nulls. In variants 4–6, null distribution of pathway activity measure alone was generated by random permutation of gene labels and *P*-value estimates were calculated using Student's *t*-test between the distribution observed in the given dataset and that in randomized gene sets. Furthermore, in variants 1 and 4, *P*-values from self-contained and competitive tests were independently combined first using Stouffer's *Z*-score method and then using the larger value of the two as the final *P*-value for a pathway (in this scenario, a pathway would score only when *P*-values from both tests would be below the significance threshold). In variants 2 and 5, *P*-values from all tests were directly combined into a single overall value using Stouffer's *Z*-score method. In variants 3 and 6, independent *P*-values were directly compared as is and the largest of the values, across both self-contained and competitive tests, was assigned as the final *P*-value for a pathway (in this scenario, a pathway would score only when deemed significant by all tests independently).

**Figure 2.**
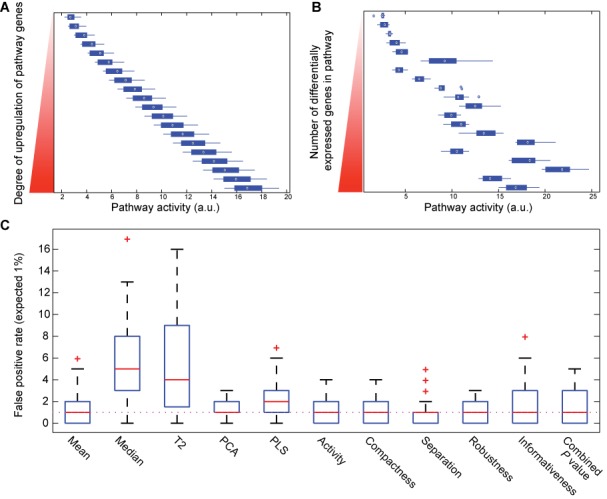
Sensitivity analysis using simulated datasets and models. (**A**) Pathway activity measure changes linearly with uniform change in expression of all annotated genes. (**B**) Pathway activity measure is linearly sensitive to change in number of significantly differentially expressed genes in a pathway. (**C**) Median value of false positive rates for all MIPA measures matches the expected rate of 1% (dotted line). Performance of MIPA measures is comparable with that of Mean and PCA methods. *P*-values from all 5 MIPA measures were combined using Stouffer's *Z*-score method.

**Figure 3.**
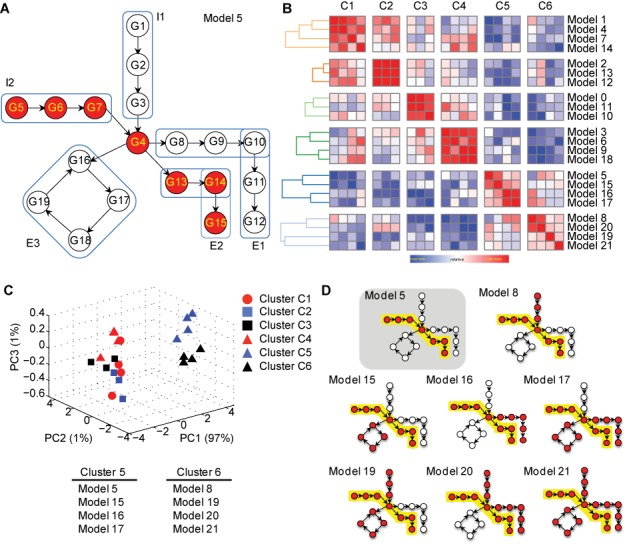
(**A**) A model pathway comprising of two influx arms (I1, I2) and three efflux arms (E1, E2, E3). (**B**) Pairwise similarity matrix, subject to hierarchical clustering, identifies six distinct clusters. (**C**) PCA-based clustering of pathway activity measures segregates models into two distinct groups, one that contains models from clusters 5 and 6 and another that contains the remainder of the models. (**D**) Models in clusters 5 and 6 include the reference model 5 and all other instances when the flux through influx arm I1 and efflux arm E2 is preserved.

**Figure 4.**
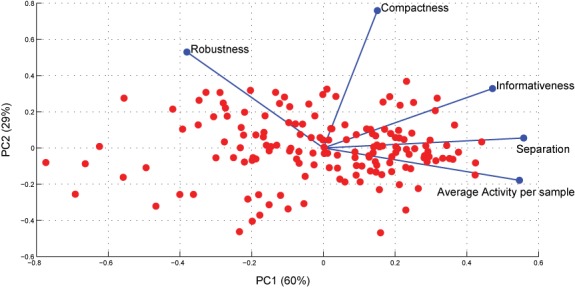
PCA analysis of matrix of MIPA measures for 156 KEGG pathways. Relative impact of each MIPA measure on overall assessment of significant pathways is presented in Table 1.

**Figure 5.**
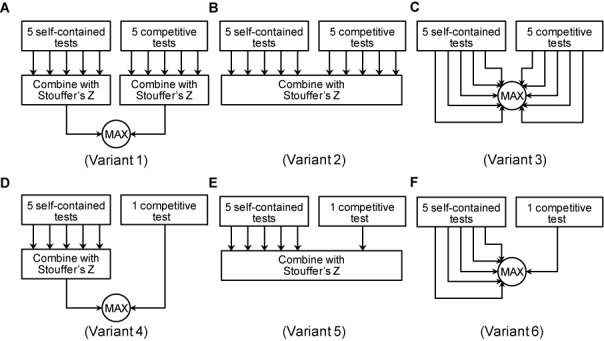
Six variants of MIPA approach. In Variants 1–3 (panels **A**, **B**, **C**), competitive tests evaluate all five measures, just like self-contained tests. Variants 4–6 (panels **D**, **E**, **F**) use a single *t*-test between the distribution of pathway activity measures observed per sample in the given dataset and that in randomized gene sets. Variants 1 and 4 (panels A, D) combine *P*-values using Stouffer's *Z*-score method and then use the larger value of the two as the final *P*-value for a pathway. Variants 2 and 5 (panels B, E) directly combine the *P*-values from all tests into a single overall value using Stouffer's *Z*-score method. Variants 3 and 6 (panels C, F) assign the largest *P*-value as the final value for a pathway.

### Datasets

#### Wound-associated epithelial transcriptional data analysis

Whole genome transcription in normal epithelium and wound-associated epithelium were profiled at days 2, 4 and 6 after colonic mucosal injury. The data was generated using Agilent's whole mouse genome microarray 4 × 44K platform. Raw data was log_2_ transformed, subject to LOESS smoothing and quantile normalized. Gene expression data was aggregated from multiple probes per gene using a maxMean function. Differentially expressed genes were identified using three methods: (i) *t*-test and fold-change at each time-point. Nominal *P*-values were estimated by a *t*-test assuming unequal variance. Significant *P*-values were selected for controlling FDR at 0.05 using a 2-stage Benjamini, Krieger and Yekutieli multiple hypothesis test correction method (17). 96 significant genes were identified on day 2, 589 significant genes on day 4 and 596 significant genes on day 6. Genes that scored as significant in at least two time points with absolute fold-change >2 were selected. (ii) ANOVA on fold-change across time. Nominal *P*-values were estimated by a one-way ANOVA on fold-change values across three days. Significant *P*-values were selected for controlling FDR at 0.05 using the same methods as above. Genes with absolute differences in fold-change > 2 in at least two time points were selected. (iii) Marker selection. Top 100 features were identified based on signal-to-noise ratio using GSEA software. In total, 914 significant genes were identified (Supplementary Table S6).

#### *Salmonella* infection in mice deficient in autophagy

Protocols for bacterial growth and infection were performed as previously described, with slight modification (18). The naturally streptomycin resistant *Salmonella enterica* serovar Typhimurium SL1344 were grown for 8 h at 37°C in LB broth supplemented with ampicillin (50 μg/ml) and subsequently diluted 1:1000 and cultured overnight under mild aeration. Bacteria were washed twice in cold phosphate buffered saline and resuspended at 10^8^ CFU/50 μl. The *S.* TYPHIMURIUM strain used in this study has been engineered to express a dsRed fluorescent protein and is ampicillin resistant (19). Water and food were withdrawn 4 h prior to treatment with 20 mg streptomycin in 75 μl; immediately following streptomycin treatment, water and food were again provided. Twenty hours later, water and food were again removed for 4 h, after which mice were infected with 10^8^ CFU *S.* Typhimurium. Water was resumed immediately and food was provided 2 h post-infection. Mice were sacrificed via CO_2_ asphyxiation at indicated time points and tissues were harvested for RNA extraction.

#### Gene silencing and PBMC stimulation for *Candida* infection study

Peripheral blood mononuclear cells (PBMC) were isolated from healthy volunteers by Ficoll-Paque gradient. After isolation 0.5 × 10^6^ cells were plated into 96 round bottom well-plates and left for 2 h at 37°C to subsequently transfect them with 25 nM NT5C3, CD38 siRNA (on target) or scrambled (non target) control siRNA (smartpool, Thermo Scientific) for 48 h at 37°C (Dharmafect, Thermo Scientific). After a centrifugation step (8 min 1800 rpm), supernatant was discarded and cell pellet was resuspended in 200 μl of RPMI (Dutch modified) containing *Candida albicans* blastoconidia or *Candida* hyphae (both UC820, 1 × 10^6^/ml, heat-killed at 100°C for 1 h) for 24 h. Supernatants were collected and IL-6 was subsequently measured by enzyme-linked immunosorbent assay (ELISA) (Sanquin Research, Amsterdam).

## RESULTS

### Multivariate pathway activity measures demonstrate linear sensitivity to transcriptional regulation of annotated genes

We examined the sensitivity of our metrics with respect to changes in degree and extent of significantly expressed genes within a pathway using simulated datasets. To compare our method with other activity inference schemes, five other expression summarization methods were implemented including mean and median approaches (used in Guo *et al.* (5)), multivariate Hotelling's *T*^2^ statistic (developed by Lu *et al.* (14)), PCA (similar to that used in Bild *et al.* (20)) and partial least squares (PLS) regression (adapted from Liu *et al.* (21)).

Two sample groups of equal size were simulated from *p*-dimensional normal distributions *N*(*μ*_1_,*Σ*_1_) and *N*(*μ*_2_,*Σ*_2_) representing two biological conditions with different outcomes (such as control versus treatment), for a pathway with *p* number of genes. To test the effect of decreasing magnitude of transcriptional regulation, 20 distinct instances of the simulated dataset were created by varying parameter α, which controlled the average fold-change in expression of all member genes. Parameter α was varied from ∼7-fold to ∼1.15-fold. To test the effect of decreasing extent of transcriptional regulation, 20 distinct instances of the simulated dataset were created by varying parameter ϒ, which determined the number of significantly upregulated genes. Parameter ϒ was varied from 19 to 0. Sensitivity was defined as the degree of change induced in the activity measure for a unit change in the independent parameter (α or ϒ). Graphically, sensitivity represents the slope of a graph plotting the change induced in activity measure versus the change in the independent parameter.

We found our pathway activity measure to be sensitive to changes in both parameters α and ϒ (Figure 2A and B), and it demonstrated a linear relationship with the changes in both parameters (Supplementary Figures S1 and S2). Between the two parameters, our primary metric was more sensitive to the change in degree of transcriptional regulation (parameter α). Among other measures, *representativeness* was most sensitive to changes in both parameters while *compactness* and *robustness (stability)* were the least responsive to parametric changes. In comparison, other FCS methods also exhibited a fairly linear relationship but were either extremely sensitive, such as Hotelling's *T*^2^ statistic (Supplementary Figures S3 and S4), or not sensitive at all, such as the PLS method. Both mean and median were responsive to changes in parameters α and ϒ, but the degree of change induced was less than moderate.

### False positive rates of MIPA metrics are within an acceptable range and comparable to that of PCA and mean methods

We analyzed the false positive rates of our metrics, as well as other methods, using another set of simulations in Matlab. The simulated datasets contained four gene sets with 5, 10, 20 and 50 genes respectively. Expression of 85 genes for two groups was generated from a multivariate normal distribution with mean vector *μ* and a diagonal variance-covariance matrix Σ. In this process, 85 elements of *μ* were generated as uniform and random variables in interval (0, 10) and the 85 diagonal elements of Σ were generated as uniform and random variables in interval (0.1, 10). The off-diagonal elements of the variance-covariance matrix Σ were varied with a correlation (*r*) of 0.1, 0.3, 0.5, 0.7 and 0.9. The simulation datasets were replicated 100× in each condition. All simulation analyses compared the expression of a gene set between two groups with the same mean expression levels, each with a sample *(N*) of 10, 25 and 50 observations. We estimated permutation *P*-values based on 500 permutations of samples in each condition. For our multivariate activity metrics, we also estimated an overall *P*-value by combining the *P*-values using Stouffer's *Z*-score method. False positive rates were estimated by the observed proportion of replicates with a *P*-value <0.01 and 0.05.

Supplementary Table S1 shows the false positive estimates, at an expected rate of 1%, for all tests for correlations 0.1, 0.3, 0.5, 0.7 and 0.9; gene set sizes of 5, 10, 20 and 50, and sample sizes of 10, 25 and 50 in each group. Overall, the median false positive rates for our metrics in these simulated datasets are at the expected values of 1 and 5% (Figure 2C and Supplementary Figure S5). In comparison to other FCS methods, the performance of our method is comparable to that of PCA and Mean algorithms, and three of the other metrics, namely Hotelling's *T*^2^ statistic, Median and PLS had significantly larger false positive rates than expected.

### Pathway activity measure is uniquely sensitive to changes in correlation structure between member genes

We next hypothesized that significant changes in gene–gene correlation structure can be informative of topological changes in the regulation of a pathway. This could be especially relevant when flux through a metabolic pathway may be re-routed between two alternate terminal end points. A pathway comprised of two influx arms (I1, I2) and three efflux arms (E1, E2 and E3) (Figure 3A), was modeled to test whether our method was sensitive to such changes. Using this specific topology, 22 distinct datasets were created to simulate different scenarios of flux re-routing. These represented models varying from no regulation (Model 0) to a fully upregulated pathway scenario (Model 21) (Supplementary Figure S6). Model 5 (Figure 3A), representing the state in which influx arm I2 and efflux arm E2 are upregulated, was selected as the reference model for basal state. Our method was tested to examine whether it could discriminate between instances when this reference flux pattern was conserved versus when it was not. Pathway activity levels were first computed for the reference state and using this ‘learned’ response model, activity levels were estimated for the other 21 states. A pairwise similarity matrix was computed using the quantitative measures of all models, and hierarchical clustering identified six distinct groups (Figure 3B; spearman rank correlation with complete linkage). Using PCA-based clustering on pathway activity measures, groups 5 and 6 clustered together and were distinct from the remainder of the models (Figure 3C). A closer evaluation of all the models from clusters 5 and 6 revealed that these clusters indeed represented scenarios in which arms I1 and E2 were upregulated along with one or more other sub-arms (Figure 3D). In comparison, none of the other FCS methods could discriminate between distinct modes of pathway regulation (Supplementary Figure S7). We could not test Hotelling's *T*^2^ statistic in this analysis because that statistic yields a single value for multiple samples within a group, whereas our method estimates activity induced per stimulated sample. This theoretical analysis confirmed that our method can uniquely discriminate between instances when a flux/signal is conserved versus when it is significantly re-routed within the same pathway.

### MIPA measures are independent and each contributes significantly to overall assessment of pathway analysis

To assess whether our multivariate pathway measures are correlated and whether all parameters contribute equally to discriminate between pathways, we examined a transcriptional dataset profiling the response of human dendritic cells to *Mycobacterium tuberculosis* (MTB) (22). Barreiro *et al.* characterized the transcript expression levels in primary dendritic cells from 65 individuals, before and after infection with MTB, generating more than 250 microarray samples. We selected this study because it provides a large sample size for a pathogen-specific transcriptional response in a single primary cell type, making it ideal for correlation and robustness analysis.

We obtained the log_2_ normalized expression matrix directly from the Gene Expression Omnibus (GEO) (GSE34151) and processed it with our pathway activity computation pipeline. We computed a matrix of MIPA measures for 156 KEGG pathways and subjected it to a principal components analysis. As shown in Figure 4, a bi-plot of the loading coefficients for all measures and principal components scores for each pathway, the first two components account for considerable fractions of variance independently (∼60% in first component, ∼29% in second component). Additionally, the loading coefficients are considerably different for each measure between the first two components, and each measure dominates uniquely in one of the five principal components (Supplementary Table S2). We also computed pairwise linear correlation coefficients between these measures and found all to be less than 0.6 (Supplementary Table S3) with the exception of *Activity* and *Separation* which was 0.82.

To further assess the contribution of each of the five parameters to the overall/final assessment of pathway significance, we computed the number of pathways that scored significantly by MIPA, first by using all five features, and then by removing one feature at a time (Table 1). We also evaluated whether the *quality* of the *P*-values estimated was significantly affected. We compared the *P*-values of the pathways between those estimated with all five measures versus ones obtained by removing just one feature using a single sample *t*-test with the null hypothesis that the differences would be centered on zero with unknown variance. As shown in Table 1, we found 57 pathways that scored significantly when using all five features. Exclusion of *Activity* and *Separation* measures had the most significant impact on the number of pathways that scored, and the results clearly indicate that loss of either of these one measures was not compensated by the other. Likewise, exclusion of all three other measures also resulted in either a loss or gain of number of significant pathways. Notably, exclusion of the *Robustness* parameter yielded almost twice as many significant pathways. To further assess the underlying cause for this result, we evaluated the PCA clusters for each additional pathway that scored. We found that each of these pathways exhibited large intra-group variance coupled with distinct sub-clusters within the phenotypically similar groups of control and stimulated samples (data not shown). Such a clustering phenomenon is deemed unstable by the *Robustness* measure and hence these pathways did not score with the original MIPA analysis of this dataset. We did not find any basis for technical noise or batch-specific differences in normalized gene expression data, which can typically manifest in large cohort studies, and which could explain this sub-clustering phenotype. We hypothesize that the true source of this phenomenon might be traceable to either demographic differences (e.g. age, gender, ethnicity) or genotypic differences within this cohort (e.g. genotypic differences resulting in eQTLs that lead to differential activity patterns within a pathway). If the latter were true, this finding would suggest that our pathway activity measure might have utility in detecting pathway-response-QTLs. These results collectively suggest that all MIPA measures are independent and each contributes significantly to overall assessment of pathway analysis.

**Table 1.** Relative impact of each MIPA measure on overall assessment of significant pathways

Feature removed	# Significant pathways	*t*-test to assess significant differences in estimated *P*-values of pathways
None	57	N/A
Avg. activity per sample	11	4.48E-42
Compactness	48	1.18E-13
Separation	11	1.15E-37
Robustness	92	1.81E-45
Informativeness	65	8.51E-07

Each of the MIPA measures were removed independently, one feature at a time, to assess the impact on the number of pathways being scored significantly relative to the original assessment when none of the features were removed. Quality of the significance profile was also assessed by single sample *t*-test of differences in log-transformed *P*-values.

### Comparative analysis of MIPA with other FCS methods benchmarks its performance and false positive rate at less than 1%

Formal error rate estimation for pathway analysis methods has been usually stymied by lack of gold standards. Typical validation approaches include citations to supporting evidence either from existing literature or from results of gene set enrichment analysis (GSEA) (23). However, a recently published study presented a systematic approach for assessment of gene set analysis methods in terms of *Sensitivity*, *Prioritization* and *Specificity* (24). Briefly, the framework proposes that any gene set analysis method, when applied to a disease-specific microarray of control and disease samples, should score the corresponding disease-relevant pathway among the top pathways. Subsequently, each method is evaluated in its ability to (i) produce small *P*-values for the relevant gene sets (*Sensitivity*), (ii) rank close to the top these context-relevant gene sets (*Prioritization*) and (iii) not generate more false positive rates than expected (*Specificity*). Their analysis identified Pathway Level Analysis of Gene Expression (PLAGE), GLOBALTEST and Pathway Analysis with Down-weighting of Overlapping Genes (PADOG) as the best overall gene-set analysis methods.

PLAGE (12) decomposes gene expression variance in each gene set using singular value decomposition. GLOBALTEST (25) uses a logistic regression model to determine if samples with similar profiles have a similar phenotype. PADOG (26) computes a gene set score as the mean of absolute moderated *t*-scores weighed inversely with the gene frequency across all gene sets analyzed. We benchmarked MIPA against these previously published performance results of other gene set analysis methods.

We obtained the normalized microarray datasets for 42 studies, totaling to >1400 samples, from Tarca *et al.* We also received gene set annotations for 259 KEGG pathways and 88 Metacore Disease Biomarker Networks. Each dataset studied one of 19 unique conditions/diseases for which KEGG or Metacore had designed a specific gene set. For each dataset independently, we first applied MIPA to estimate the *P*-value for all gene sets and sorted them from lowest to highest *P*-value. We then computed the rank of the target gene set (specific to that disease dataset) as a percentage ranging from ∼0 to 100% (small rank values would indicate that the target gene set was prioritized as relevant). Next, we ran our method on 50 phenotype permuted versions of each of the 42 datasets as outlined earlier (24). We counted the number of gene sets with a *P*-value <0.01 and 0.05 under this null hypothesis and expressed this number as a percentage of the total number of tests.

In this comparative analysis, we analyzed the performance of six different variants of MIPA (outlined in ‘Materials and Methods’ section; Figure 5). Table 2 summarizes the performance parameters for each of these six variants. For a given competitive test of choice, we found that the approach of combining *P*-values using Stouffer's *Z*-score method resulted in higher sensitivity than simply taking the largest of the *P*-values across all tests. However, combining *P*-values directly (variants 2 and 5) resulted in very large false positive rates. Variants 1 and 4, both of which use the approach of combining *P*-values separately first, performed extremely well on all parameters, with variant 4 achieving false positive rates of less than that expected at random (1%).

**Table 2.** Comparative performance of six variants for MIPA in their ability to (i) produce small *P*-values for the relevant gene sets (*Sensitivity*), (ii) rank close to the top these context-relevant gene sets (*Prioritization*) and (iii) not generate more false positive rates than expected (*Specificity*)

Method	*P*-value (sensitivity)		% Rank (prioritization)		False positive rate (specificity)	
	Median	Mean	Median	Mean	1%	5%
MIPA_var_1	0.0025	0.2682	23.5812	30.9202	2.5427	5.0149
MIPA_var_2	0.0000	0.1132	3.3994	22.2025	14.2291	19.2215
MIPA_var_3	0.9200	0.8329	79.4737	73.3134	0.0000	0.0000
MIPA_var_4	0.0299	0.3079	44.2210	47.2948	0.7354	1.1496
MIPA_var_5	0.0000	0.0777	6.2500	25.1189	27.9256	36.4886
MIPA_var_6	0.7900	0.7152	64.1938	61.3367	0.0000	0.0001

We compared the performance scores of our method (variants 1 and 4) with those already reported for 16 other FCS methods (Table 1 in (24)). We re-normalized the prioritization score and sensitivity surrogate into *Z*-scores after adding data for our method. Previous analysis segregated the methods into two categories, based on the false positive rates observed with each method, and generated separate rankings for the two. Category 1 methods generated the expected (or close to expected) number of false positives whereas Category 2 methods had much larger false positive rates under the null hypothesis. Our method, MIPA (variants 1 and 4), clearly would be classified as Category 1 based on their *Specificity* profiles. The previous study did not use *Sensitivity* data when ranking the methods in Category 1. We produced two overall rankings, one with *Specificity* incorporated abnd the other without. Supplementary Table S4 summarizes these overall rankings. Variant 1 of MIPA was the top-scoring method and outperformed all other methods in the comparison. Variant 4 of our method, which has a better *Specificity* profile than variant 1, ranked second after PLAGE but has a superior false positive rate.

Finally, we examined the *Robustness (or Reproducibility)* of our method as considered by Li *et al.* (27). We utilized the transcriptional dataset from Barreiro *et al.* (22) and derived 50 resampled subsets by randomly selecting 78 paired samples, with <50% sample overlap between the subsets. We counted the number of gene sets that scored with MIPA (*P*-value < 0.01). We found that in spite of random resampling from the same large set, the number of gene sets called significant by MIPA did not vary greatly from one set to the other (Supplementary Figure S8). On average, ∼60 gene sets scored across this resampling analysis (standard deviation = 6 pathways). When compared to the results from applying MIPA to the full dataset, we found 57 significant pathways, of which 40 scored in at least 80% of the resampled datasets. From this resampling analysis, we conclude that MIPA is fairly robust and that we can expect the end results to be fairly reproducible and transferable across similar datasets.

### Up to 1000 iterations are sufficient for identification of driver genes

We analyzed whether 1000 permutations were enough for the identification of driver genes in pathways annotated with varying number of genes. We generated simulated datasets that contained four gene sets with 5, 10, 25 and 50 genes. Expression of genes for two groups was generated from a multivariate normal distribution with mean vector *μ* and a diagonal variance-covariance matrix Σ. The off-diagonal elements of the variance-covariance matrix Σ were varied with a correlation of 0 to 0.5 and the difference between the means of two groups was set to 5. We applied our driver gene identification algorithm to each of these gene sets and varied the number of allowed iterations from 100 to 4000 in steps of 100. Each iteration produced a rank-ordered list of genes per pathway, which was saved to build a single matrix of rankings assigned to each gene across all runs. Next, we computed the Spearman rank correlation coefficient between each ranking from a run and the ranks obtained at the final run with 4000 iterations. As shown in Supplementary Figure S9, the correlation coefficient increases asymptotically and typically exceeds 0.8 in runs with as few as 500 iterations. This result illustrated that up to 1000 iterations were sufficient to use in the identification of driver genes per pathway.

### Higher sensitivity of MIPA identifies pathways not enriched in GSOA and GSEA

Based on the outcome of our sensitivity analysis on simulated data and comparative performance analysis, we hypothesized that our method would be able to detect regulation even in the presence of small coordinated transcriptional change across several genes in a pathway. This would be particularly advantageous in detecting instances when small regulation of a critical gene, such as a bottleneck enzyme in a metabolic pathway or an adaptor molecule for a signaling complex, would have significant impact in the output of the pathway. The efficacy of our method was tested using transcriptional profiles of intestinal injury and wound healing (28). Seno *et al.* developed an *in vivo* acute injury system, analogous to punch biopsy of the skin, which allowed them to generate temporal profiles of intestinal epithelial injury as it progressed through the various phases of healing. They showed that Trem2 was essential to promote healing of wounds in the colon. We applied our method to identify pathways that are differentially regulated during this wound healing process.

After initial normalization and pre-processing (described in ‘Materials and Methods’ section), the transcriptomics dataset was subject to our pathway activity analysis pipeline. Our method identified 49 pathways as significant at FDR < 0.25 (Supplementary Table S5). These included several of the well-known pathways and processes that are regulated in wound-healing response such as the cytokine–cytokine receptor interactions and growth-factor driven signaling (ErbB signaling) (29). Also, the activation of pattern recognition receptors, such as NOD-like and RIG-I-like receptors that are instrumental in detecting various pathogens and generating innate immune responses, are suggested to play an essential role in maintenance of gut homeostasis (30). Other specific signaling pathways that also scored in our analysis and have been identified for their role in wound healing include MAPK, JAK-STAT and NFκB signaling (31,32).

**Figure 6.**
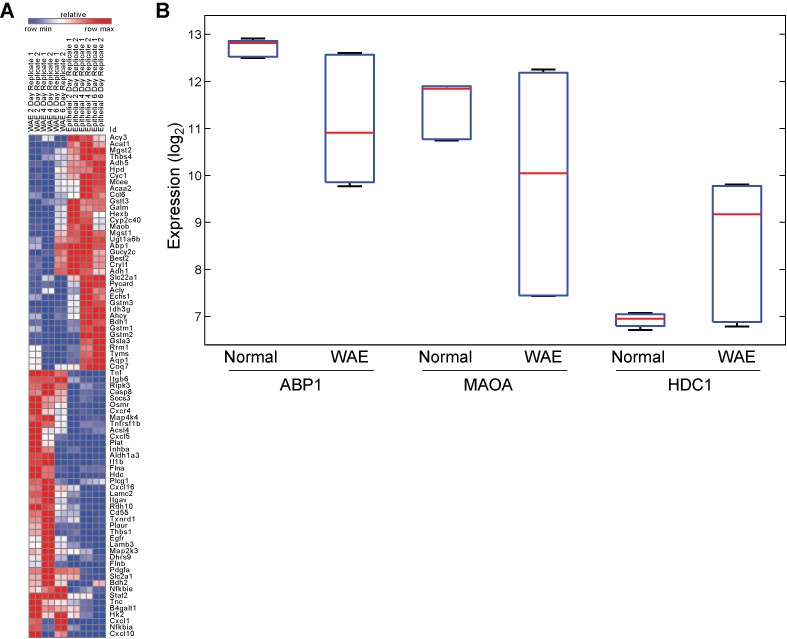
Pathway activity analysis of wound-healing response. (**A**) Seventy-nine driver genes identified from 49 pathways that scored significantly with MIPA analysis. (**B**) Differentially regulated driver genes of histidine metabolism.

We next identified 643 *driver* genes for the set of 49 pathways (Supplementary Table S5), 79 (12.3%) of which showed locally significant differences between the two tissue groups (ANOVA, FDR < 0.05; Figure 6A). A hyper-geometric test was used to rank-order pathways based on the enrichment of *driver* genes that scored as significantly differentially expressed at the whole-genome level. Four pathways scored with significant *P*-value < 0.05, which included signaling pathways of cytokine receptors and growth factors, and metabolic pathways of glutathione and histidine metabolism (Table 3). Collectively, these pathways and their select driver genes represent suitable targets for functional validation. Histidine metabolism, in particular, has been shown to accelerate the wound-healing process (33) and ameliorate murine colitis (34). Our analysis identified HDC1, ABP1 and MAOA as the significant driver genes in this pathway (Figure 6B). Using network-building algorithms in MetaCore (Thomson Reuters; Ref: http://thomsonreuters.com/metacore/), we identified regulatory paths from these driver genes to Trem2 (Supplementary Figure S10). Our model suggests that collective up-regulation of HDC1 and down-regulation of ABP1 and MAOA, lead to increased accumulation of histamine, which activates Trem2 and accelerates the wound healing process.

**Table 3.** Top-scoring wound healing response pathways and key driver genes identified by MIPA

Pathway	MIPA (*P*-value)	Driver genes	GSEA	GSOA
Glutathione metabolism	0.0182	*Gstm1, Gstm2, Gstm3, Gsta1, Gsta3*	Down	Down
Cytokine-cytokine receptor interaction	0.0151	*Tnfrsf11a, Inhba, Il1b, Cxcl10, Cxcl5, Egfr, Cxcl16, Cxcr4, Osmr*	Up	Up
ErbB signaling pathway	0.0479	*Erbb4, Hbegf, Cdkn1a, Egfr*	Up	Up
Histidine metabolism	0.0455	*Abp1, Maoa, Hdc*	N.D.	N.D.

We compared the performance of our method with two other popular methods for identifying significant pathways, namely, gene set overlap analysis (GSOA)(35) and GSEA (23). The two methods identified very distinct signatures of pathways with overlap as little as 20%. GSEA identified 40 gene sets as significantly enriched (*Q*-value ≤ 0.25, Supplementary Tables S7 and S8), while GSOA identified 49 enriched gene sets (*Q*-value < 0.05, Supplementary Tables S9 and S10) on the basis of 914 significantly differentially expressed genes (Supplementary Table S6). Comparatively, our method identified 25 pathways that were not reported by either GSEA or GSOA. These included several well-known processes in wound-healing response such as NFκB signaling and RLR/NLR signaling. Histidine metabolism, identified as one of the key pathways for the wound healing process by our method, did not score as significant with either GSEA or GSOA. As shown in Table 4, most of these pathways had either none or just a few significantly differentially expressed genes, which explains why these were not enriched. This observation clearly illustrated that our method was indeed able to detect pathways already known to be associated with wound healing, even when there was low transcriptional regulation of annotated member genes.

**Table 4.** Wound healing response pathways uniquely identified by MIPA

Path	# Annotation genes	# Driver genes	# Sig.diff.genes	Min rank SNR	Mean rank SNR	Median rank of genes	Max rank SNR
Pentose and glucuronate interconversions [Path:mmu00040]	27	17	1	2974	9833.75	11119	12969
Synthesis and degradation of ketone bodies [Path:mmu00072]	9	6	0	164	9381	10206.5	13030
Ubiquinone and other terpenoid-quinone biosynthesis [Path:mmu00130]	10	7	1	2863	9709.2	11022.5	13104
Purine metabolism [Path:mmu00230]	151	14	0	68	7549.297297	8185.5	13011
Histidine metabolism [Path:mmu00340]	28	11	3	7	8406.884615	10023	13052
Phenylalanine, tyrosine and tryptophan biosynthesis [Path:mmu00400]	7	6	0	2863	7231.8	6535	12654
Cyanoamino acid metabolism [Path:mmu00460]	4	2	0	920	9397.5	11898.5	12873
N-Glycan biosynthesis [Path:mmu00510]	47	5	1	368	8320.069767	9924	12896
Glycosaminoglycan degradation [Path:mmu00531]	18	9	1	1280	7398.176471	7623	13057
Sphingolipid metabolism [Path:mmu00600]	38	14	0	116	6852.361111	7554	12667
Glycosphingolipid biosynthesis—globo series [Path:mmu00603]	14	9	1	640	7690.928571	8637	13057
Glyoxylate and dicarboxylate metabolism [Path:mmu00630]	22	6	0	4254	10423.14286	11506	13065
Butanoate metabolism [Path:mmu00650]	27	5	0	164	9840.72	11173	13095
One carbon pool by folate [Path:mmu00670]	16	9	2	1344	7574.25	7336	13100
Riboflavin metabolism [Path:mmu00740]	12	8	0	3563	8754.333333	8901.5	12512
Retinol metabolism [Path:mmu00830]	63	38	4	38	7639.296296	7795	12975
NF-kappa B signaling pathway [Path:mmu04064]	84	13	0	14	4212.3125	2738	12849
Phagosome [Path:mmu04145]	150	26	1	46	4590.588785	3630	13107
NOD-like receptor signaling pathway [Path:mmu04621]	47	8	0	14	5028.02381	3771	13145
RIG-I-like receptor signaling pathway [Path:mmu04622]	59	11	1	24	4681.5	3239	12736
T cell receptor signaling pathway [Path:mmu04660]	106	27	1	97	4902.640777	3850	12928
Adipocytokine signaling pathway [Path:mmu04920]	66	16	2	87	5438.153846	5423	12745
Salivary secretion [Path:mmu04970]	70	28	1	589	5852.044776	5043	12497
Bile secretion [Path:mmu04976]	65	29	2	271	6512.473684	6275	13131
Vitamin digestion and absorption [Path:mmu04977]	21	14	1	634	6075.142857	6180	12390

### MIPA identifies novel targets of *Atg16l1* during response to *Salmonella* infection

Our group recently demonstrated that loss of expression of the essential autophagy protein Atg16l1 in intestinal epithelial cells decreased autophagic engulfment of S. Typhimurium, resulting in hyper-inflammation and systemic bacterial translocation in infected mice (36). To gain further insight into other pathways and genes that are regulated by Atg16l1, we generated transcriptional profiles of ileal tissue from wild-type mice and mice deficient in autophagy in epithelial cells (*Atg16l1^f/f^* x Villin-cre) or Cd11c^+^ cells (*Atg16l1^f/f^* x Cd11c-cre), after *in vivo* infection with *Salmonella*.

**Figure 7.**
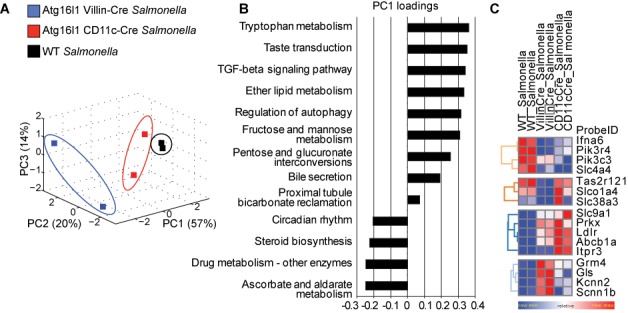
Identification of gene–gene interactions for Atg16l1. (**A**) PCA-based clustering of inferred activity for 13 pathways regulated in response to *Salmonella* infection in intestinal epithelial cells. (**B**) Pathway activity loading coefficients along the first principal component. (**C**) Driver genes that were significantly dysregulated in *Atg16l1^f/f^* x Villin-cre samples.

After initial pre-processing and normalization of transcriptional data (see ‘Materials and Methods’ section), our method identified 13 pathways regulated in response to *Salmonella* infection in wild-type intestinal epithelial cells. These included regulation of autophagy, the TGFβ signaling pathway, steroid biosynthesis and several metabolic pathways (Supplementary Table S11). The induced activity levels for these select pathways were cross-compared with those predicted for autophagy-deficient mice, and were subjected to PCA-based clustering. These 13 pathways formed a robust basis to discriminate between wild-type and autophagy-deficient groups (Figure 7A). The response to *Salmonella* infection from *Atg16l1^f/f^* x Cd11c-cre mice was similar to that observed in wild-type mice, but the samples from *Atg16l1^f/f^* x Villin-cre mice were clearly distinct from both of the other groups. These results are consistent with our *in vivo* analysis of these mice, in which we observed that *Atg16l1^f/f^* x Villin-cre had increased systemic inflammation following infection with *S.* Typhimurium, but that there were few such differences between *Atg16l1^f/f^* x Cd11c-cre and wild-type mice. Amongst these 13 pathways, we evaluated the loading coefficients along the first principal component, which accounted for >50% variance in the data, and could readily discriminate between the three groups. We found that activity measures from pathways of tryptophan metabolism, taste transduction and TGF-ß signaling contributed most significantly to the observed separation between sample groups (Figure 7B). Two of these pathways have recently been shown to be associated with autophagy. Wauson *et al.* (37) have demonstrated that reduced expression of taste receptor complex T1R2/T1R3 accelerates autophagy while Fougeray *et al.* have reported that depletion of tryptophan mediates activation of IFN-ϒ-induced autophagy (38).

Next, we identified the *driver* genes for this set of 13 significant pathways and compared the differences in their expression fold-change between wild-type and autophagy-deficient samples (ANOVA; *P*-value < 0.05) to identify 16 genes that were significantly dysregulated in *Atg16l1^f/f^* x Villin-cre samples (Figure 7C). These included previously reported regulators of autophagy, namely *Ifna6*, *Pik3r4* and *Pik3c3*. The other notable genes included members of the bile secretion pathway (*Slc4a4*, *Slc9a1*), gene *Gls* from the bicarbonate reclamation pathway and *Tas2r121* from the taste transduction pathway. *Tas2r121* belongs to the family of bitter taste receptors that are being increasingly recognized for their prominent role in ‘sensing’ the luminal content of mucosal surfaces and affecting gastrointestinal function and glucose metabolism (39). In a recent report Lee *et al.* provide evidence that these receptors also function as regulators of innate immunity and antimicrobial defense in the human upper respiratory tract (40). Such cell type-specific autophagy-dependent regulation of pathways may have important implications for host response to infection.

### Comparative MIPA identifies nicotinamide metabolism specifically required for anti-fungal response

In a recent report, type 1 interferon signaling was recently identified as a critical pathway for host defense against *C. albicans* (41). Using a whole-genome transcriptional dataset of human primary cells stimulated *in* vitro with bacterial and fungal agents, the previous bioinformatics approach focused on analysis of signaling pathways (MetaCore) and reported interferon signaling to have the strongest enrichment within this set. We sought to use our newly developed approach to identify metabolic pathways that may be additionally regulated.

Normalized transcriptional profiles of 300 peripheral blood mononuclear cell (PBMC) samples were sourced from healthy individuals, at 4 and 24 h following stimulation with five different Toll-like receptor (TLR) ligands and microbes (*C. albicans, Escherichia coli*-derived lipopolysaccharide (LPS), *Borrelia burgdorferi*, MTB, or control RPMI culture medium). Application of our method to each stimulus and time point separately yielded distinct sets of pathway signatures for LPS, MTB, *Borrelia* and *Candida* response at 4 and 24 h (Supplementary Tables S12–S19). Pathways with significant *P*-values were corrected to control for FDR < 0.1 using a 2-stage Benjamini, Krieger and Yekutieli multiple hypothesis test correction method (17). Stimulation by *Candida* resulted in activation of 60 pathways at 4 h post-stimulation, which was more than twice the number of pathways, on average, that were active at the same time point in response to LPS, MTB or *Borrelia*. A comparative gene expression analysis yielded a similar trend for the number of differentially expressed genes for each stimulus (data not shown). To identify stimulus-specific pathways, a matrix of activity measures from all sample groups was assimilated and subjected to a one-way ANOVA. We selected 44 pathways with significant *P*-values corrected for FDR < 0.1 (Supplementary Figure S11A).

**Figure 8.**
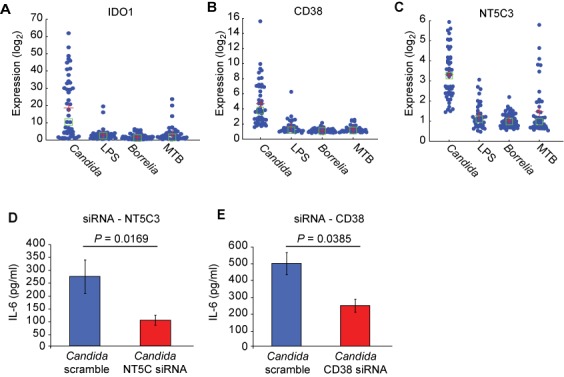
Identification of gene–environment interactions in host immunity. (**A**,**B**,**C**) Gene expression of select driver genes identified in tryptophan metabolic pathway (IDO1) and nicotinamide metabolism (CD38, NT5C3). (**D**,**E**) Effect of knocking down CD38 and NT5C3 on IL-6 production in response to *ex vivo* stimulation of PBMCs from healthy individuals with *Candida* conidia.

Using the *driver* gene identification algorithm coupled with an ANOVA test, pathway- and stimulus- specific genes were identified at FDR < 0.01 (Supplementary Figure S11B). GSOA of this list of key driver genes, using canonical pathway annotations from Reactome and Biocarta in mSigDB, yielded a strong enrichment for interferon signaling, as was previously reported (Supplementary Table S20). Four of the top 10 genes in this list were validated in the original study, namely *IFNG*, *CCL8*, *CXCL10* and *ISG15*. Another noteworthy driver gene in this list was *IDO1* from the tryptophan metabolism pathway (Figure 8A). We and others have previously reported data (42) validating *Candida*-specific upregulation of *IDO1* and the role of tryptophan metabolism in host defense against *Candida*. To prioritize among the 44 selected pathways for functional validation, we evaluated which of these pathways were enriched for the set of stimulus-specific driver genes identified in the last step. We used a hypergeometric test to assess the significance of each pathway and adjusted these *P*-values with the Benjamini-Hochberg method to control for FDR < 0.25. Ten pathways scored (Table 5), most of which were well-studied signaling pathways in the context of stimulation by TLR ligands. Nicotinamide metabolism pathway was a novel metabolic pathway that scored in this list with significant upregulation of two driver genes, *CD38* and *NT5C3*, specifically in context of stimulation by *Candida* (Figure 8B and C). Nicotinamide metabolism acts downstream of the tryptophan metabolic pathway and leads to the generation of nicotinamide from NAD+ and NADP. Based on these findings, and the results of our pathway activity analysis, we tested the hypothesis that activation of nicotinamide metabolism, via the upregulation of *CD38* and *NT5C3*, would be involved in the stimulation of immune cells specifically by *C. albicans*.

**Table 5.** Top-scoring TLR stimulation-specific response pathways identified by MIPA

Pathway	Fraction of stimulus-specific driver genes (per pathway)	Fraction of stimulus specific driver genes (background)	*P*-value	*Q*-value
Toll-like receptor signaling pathway [Path:hsa04620]	0.1164	0.0138	0.0000	0.0000
Chemokine signaling pathway [Path:hsa04062]	0.0714	0.0138	0.0000	0.0000
Cytokine-cytokine receptor interaction [Path:hsa04060]	0.0529	0.0138	0.0000	0.0000
RIG-I-like receptor signaling pathway [Path:hsa04622]	0.0750	0.0138	0.0006	0.0044
NF-kappa B signaling pathway [Path:hsa04064]	0.0723	0.0138	0.0007	0.0044
Cytosolic DNA-sensing pathway [Path:hsa04623]	0.0694	0.0138	0.0024	0.0123
Osteoclast differentiation [Path:hsa04380]	0.0333	0.0138	0.0244	0.0988
PI3K-Akt signaling pathway [Path:hsa04151]	0.0027	0.0138	0.0255	0.0988
Nicotinate and nicotinamide metabolism [Path:hsa00760]	0.0909	0.0138	0.0329	0.1134
Jak-STAT signaling pathway [Path:hsa04630]	0.0259	0.0138	0.0770	0.2388

We knocked down *CD38* and *NT5C3 ex vivo* in PBMCs isolated from healthy individuals and stimulated these cells with two distinct forms of *Candida*, namely *Candida* conidia *and* *Candida* hyphae. *C. albicans* is a dimorphic fungus that can be present in both of these two morphotypes when stimulated or activated. Twenty-four hours post-stimulation, cell cultures were profiled for interleukin (IL)-6 production, a critical cytokine required for defense against *Candida* (43). Of the eight donors used to study the effect of knocking down *NT5C3* on the response to *Candida* conidia, significant downregulation in *IL-6* production was observed in six cases (*P*-value = 0.0169; Figure 8D). In the case of infection with *Candida* hyphae, a reduction in IL-6 production was observed when *NT5C3* was knocked down; however, the results were not significant (*P*-value = 0.1858) (data not shown). Knockdown of *CD38* had a significant impact on IL-6 production in response to *Candida* conidia (*P*-value = 0.0385; Figure 8E). These experimental findings validated the hypothesis predicted by our method that upregulation of nicotinamide metabolism is essential for mounting a successful anti-fungal program in human PBMCs.

### MIPA identifies a microbiome-dependent pathway associated with therapeutic response

To test whether our pathway activity measure could serve as a basis for stratifying patients and understanding therapeutic response, we analyzed publicly available mRNA expression in mucosal biopsies taken from inflammatory bowel disease (IBD) responders and non-responders to infliximab treatment (44). Infliximab is an effective treatment for ulcerative colitis (UC) with >60% of patients responding to treatment and up to 30% reaching remission. However, anti-TNF treatments such as infliximab involve high costs of treatment and show wide variance in clinical efficacy and outcomes. Certain patients may benefit more than others, but we lack reliable predictors of treatment outcome that could help stratify patients who would benefit most from such treatment. Previous attempts to derive pathway signatures using enrichment methods of differentially expressed genes have failed to induce changes in clinical practice.

We applied our method to the transcriptional profiles of biopsy samples collected from patients with clinically active UC (Mayo score > 6). These patients were treated with infliximab or placebo at weeks 0, 2, 6 and every 8 weeks thereafter until 30 weeks; biopsies were collected at baseline and at the 8 and 30 week timepoints. The patients were stratified into four groups: infliximab responders (*n* = 14), infliximab non-responders (*n* = 15), placebo responders (*n* = 6) and placebo non-responders (*n* = 8).

**Figure 9.**
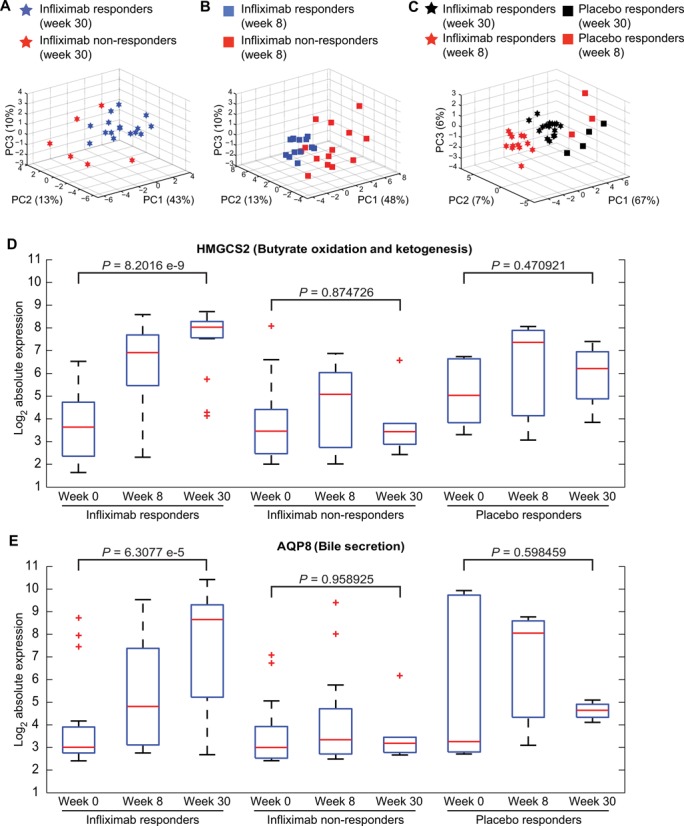
Pathway activity analysis of infliximab response in ulcerative colitis (UC). PCA-based clustering analysis of pathway activity measures for infliximab-specific pathways differentiates between infliximab-treated responders and non-responders at week 30 (**A**) and at week 8 (**B**). (**C**) Placebo-treated groups, both responders and non-responders, can be distinguished from infliximab treated groups, at weeks 8 and 30 post-treatment, using inferred activity levels of 18 infliximab-associated pathways. (**D**,**E**) Gene expression for HMGCS2 and AQP8. *P*-values were estimated using Student's *t*-test.

Our method identified 39 pathways that showed significant regulation trends in infliximab responders at week 30 post-treatment. The loading coefficients, computed within the PCA framework, formed the ‘pathway response model’ (39 models in total). Application of these models to the transcriptomics data from infliximab non-responders yielded the ‘predicted’ level of activity induced. Our hypothesis was that the pathways essential for mounting a favorable response to infliximab treatment would be predicted to have lower activity levels in the non-responder population. Comparative analysis of ‘observed’ and ‘predicted’ activity levels identified 11 pathways that were significantly upregulated in infliximab responders. PCA-based clustering analysis of the matrix of activity measures demonstrated the efficacy of these pathways in discriminating between clusters of responders and non-responders at week 30 (Figure 9A). A similar trend was observed with the transcriptomics profile at week 8 post-treatment (Figure 9B).

To identify which of the 39 pathways were specific to infliximab response, the predicted activity levels of placebo responders were analyzed in the same manner as that for infliximab non-responders. Thirty pathways were found to be shared between the two groups of responders. Combining the 9 pathways that were not regulated in placebo responders with 11 pathways not regulated in infliximab non-responders, a total of 18 infliximab response-specific pathways were identified (Supplementary Figure S12A). To test the discriminatory power of these 18 pathways, activity values from all patients, observed and predicted, were aggregated into a single matrix and subjected to PCA-based clustering. We found that 80% of variance could be explained with the first three principal components and that the four patient groups clustered distinctly from each other (Figure 9C).

To establish a molecular basis for the differences between infliximab responders and non-responders, we applied our *driver* gene identification algorithm to the 11 pathways that were predicted to be downregulated in non-responders and identified 44 genes that were differentially expressed in the non-responders. Our top-scoring gene was 3-hydroxy-3-methylglutaryl-CoA synthase 2 (*HMGCS2*) (Supplementary Figure S12B) from the ketogenesis pathway, which exhibited a >50-fold change at the end of 30 weeks of infliximab treatment in the responder group but was largely not expressed in the non-responder group (Figure 9D). Our second best scoring gene was aquaporin 8 (*AQP8*) from the bile secretion pathway, which was upregulated >50-fold in most of the infliximab responders by week 30 (Figure 9E). In comparison to the group of placebo responders, we found that the activation of these genes is uniquely associated with infliximab treatment. A *t*-test-based evaluation of the differences between expression levels before and 30 weeks after treatment yielded highly significant *P*-values of 8.2016e-9 and 6.3077e-5 for *HMGC2* and *AQP8*, respectively. Significant differences were not observed in infliximab non-responders nor in placebo responders.

HMGCS2 is a mitochondrial enzyme that catalyzes the first reaction of ketogenesis, a metabolic pathway that provides lipid-derived energy for various organs during times of carbohydrate deprivation, such as fasting. The primary substrate for this metabolic pathway is acetyl-CoA, which in turn is derived from β-oxidation of short chain fatty acids such as butyrate, when glucose is limiting. A recent study showed that *HMGCS2* expression is directly induced by butyrate in human colonic mucosa (45). Additionally, this butyrate-dependent upregulation of *HMGCS2* is driven specifically by butyrate-producing bacterial species colonized in the gut (46,47). A positive correlation between increased butyrate oxidation rates and mucosal healing for IBD patients has been demonstrated by several groups (48–50). Our group recently reported that an IBD-associated decrease in *Roseburia* and *Phascolarctobacterium* may reflect a decrease in butyrate production (51). Based on these published findings and our present results, we posited that activation of the ketogenesis pathway (by microbiome-derived butyrate via *HMGCS2*) is critical for a successful response to infliximab. Additionally, we predicted that UC patients bearing an abundant population of butyrate-producing microbiota are more likely to respond to infliximab than patients with significantly decreased abundance of these specific microbial clades. A recent report by Machiels *et al.* (39) shows evidence in close support of this hypothesis. This group reported a significant decrease in the butyrate-producing bacterial species *Roseburia hominis* and *Faecalibacterium prausnitzii* in patients with UC and concluded that restoration of these bacterial species, or the functional pathways affected by this dysbiosis, would enhance successful treatment outcomes. In addition to *HMGCS2*, several published reports provide evidence in support of *AQP8* as a candidate marker associated with successful treatment response to UC (52–54).

Our method enabled us to gain pathway-centric insights into treatment response where other approaches have failed. We stratified patients on the basis of our activity measure and generated a specific hypothesis about a microbiome-regulated host metabolic pathway that may serve as a biomarker for matching patient subsets with targeted therapy. The approach presented here resonates with the principles of ‘personalized healthcare’ model for patient stratification and stratified medicine (55).

## DISCUSSION

In this report, we present a computational method to functionally interpret high-throughput gene expression data and characterize the levels of activity induced in biological pathways. In contrast to other existing FCS methods, MIPA proposes to evaluate not one but five PCA clustering-based summary statistics. Systematic sensitivity analysis demonstrated that our metrics are linearly sensitive to changes in both magnitude and extent of transcriptional regulation. Other methods, in contrast, can be too sensitive, less than moderate, or non-responsive to changes in the same parameters.

All pathway-level aggregate statistics are significantly affected by correlation between member genes. Previous methods either employ resampling-based approaches that maintain the correlation structure of the expression data (56), or attempt to correct for these correlations by estimating a variance inflation factor directly from the data (57). However, it is conceivable that correlation between genes could provide additional information, especially when the correlation varies substantially across experiments. Such a change in correlation structure could be indicative of flux re-routing in a metabolic pathway. However, whether any of the existing methods have the ability to detect such transcriptional regulation of pathways had not been examined to date. We have shown that our method can uniquely discriminate between instances when a flux/signal is conserved versus when it is significantly re-routed within the same pathway. Our analysis of other methods showed that they failed to demonstrate such sensitivity.

The final step for any FCS method is assessing the significance of the inferred activity measure using one of the two null hypothesis: (i) a self-contained null hypothesis that permutes the sample class labels, preserves the correlation structure between genes and compares the set of genes with itself or (ii) a competitive null hypothesis that permutes gene annotations for each set and compares with a same-size set of genes that are not in the pathway. Most FCS methods typically choose one of the two methods, although it has been recommended that self-contained tests be used as an initial screening to assess the relevance of individual biological processes, followed up with a competitive test to distinguish the most important biological processes from others that are less significant (35). Following the recommended guideline, our framework incorporates a two-stage significance analysis pipeline, yielding a robust analysis.

We provide a comprehensive evaluation of every aspect of our method. We used simulated datasets to estimate the sensitivity and false positive rates of our metrics. We demonstrated that MIPA outperforms 16 other methods in terms of *Sensitivity, Prioritization* (of known targets) and *Specificity*. We present an in-depth assessment of robustness and reproducibility with our method. We believe this analytical framework can serve as a template for rigorously analyzing other methods as well.

We selected four transcriptomics datasets that profiled biological systems at varying scales of complexity and represented distinct physiological and biological states. Application of our computational framework to these case studies collectively established that our method can (a) identify regulation in pathways missed by other approaches, (b) identify gene–gene interactions in genetically engineered mouse models, (c) identify pathways at the interface of gene–environment interactions in pathogen-specific host immune responses and (d) stratify patients and generate specific hypothesis along the gene–environment-therapeutic axis.

The ability to quantify pathway activity for each sample individually facilitates comprehensive post-hoc analysis and serves as a robust basis for developing pathway-centric models and classifiers with machine-learning algorithms. The additional capability to identify a subset of *driver* genes for any given pathway, which can then be profiled on a targeted platform such as NanoString, opens up the possibility of developing pathway reporter codesets. The transcriptional profile of these reduced codesets can faithfully reproduce the pathway activity signal using our method, and can be further applied to either classify patient samples or investigate the functional impact of perturbation or genetic manipulation studies. We believe our method can not only be readily applied to other omics datasets independently but can also be extended to integrate data from diverse modalities by building a coupled annotation set (e.g. pathways annotated with genes and metabolites).

Rapid advances in sequencing technologies are ushering in an era in which generating genome-wide time-series expression data in a cost-effective manner may be possible. Irrespective of the kind of data one generates, be it transcriptomics, metabolomics or proteomics, the ultimate endpoint for all functional interpretation tasks is to understand pathway regulation. We believe that characterizing pathway dysregulation is central to understanding complex disease pathogenesis and that therapeutic interventions must be designed with an end-goal of modulating pathway activities. Our method provides a computational framework to support such systems biology research, and we present experimental evidence that corroborates its accuracy, specificity and advantages over other methods.

## SUPPLEMENTARY DATA

Supplementary Data are available at NAR Online.

SUPPLEMENTARY DATA
