# Supplementary material for: Multivariate inference of pathway activity in host immunity and response to therapeutics
Source: Nucleic Acids Res. 2014 Aug 21;42(16):10288–306. doi: 10.1093/nar/gku722 (PMC4176341; doi:10.1093/nar/gku722)
Supplement: SUPPLEMENTARY DATA [file supp_42_16_10288__index.html]

Multivariate inference of pathway activity in host immunity and response to therapeutics — Multivariate inference of pathway activity in host immunity and response to therapeutics — SUPPLEMENTARY DATA 

# Multivariate inference of pathway activity in host immunity and response to therapeutics

## SUPPLEMENTARY DATA

**Files in this Data Supplement:**

- SUPPLEMENTARY DATA
