# Supplementary material for: Multivariate inference of pathway activity in host immunity and response to therapeutics
Source: Nucleic Acids Res. 2014 Aug 21;42(16):10288–306. doi: 10.1093/nar/gku722 (PMC4176341; doi:10.1093/nar/gku722)
Supplement: SUPPLEMENTARY DATA [file supp_gku722_nar-01646-n-2014-File011.zip › NAR-01646-2014 Suppl files/Goel_Supplement_figs_text.pdf]

Figure S1. Sensitivity analysis of pathway activity measures to changes in fold change expression

Figure S2. Sensitivity analysis of pathway activity measures to changes in number of upregulated genes

Figure S3. Sensitivity analysis of other FCS methods (Mean, Median, Hotelling's T^2, PCA, PLS) to changes in fold change expression

Figure S4. Sensitivity analysis of other FCS methods (Mean, Median, Hotelling's T^2, PCA, PLS) to changes in number of upregulated genes

Figure S5. False positive error estimates in all FCS methods at an expected rate of 5%

Figure S6. Models simulating different scenarios of flux regulation within a pathway

Figure S7. PCA-based clustering of activity measures estimated from other FCS methods (Mean, Median, PCA, PLS)

Figure S8. Number of significant pathways in resampled random datasets.

Figure S9. Spearman rank correlation coefficients of gene rankings between each independent run and final run (with 4000 iterations) for analysis of driver gene identification algorithm.

Figure S10. Proposed model for regulation of Trem2 and wound healing by histidine metabolism

Figure S11. Antifungal and antibacterial response pathways and driver genes

Figure S12. Infliximab response-specific pathways and driver genes

Table S1. False positive rates estimated for MIPA measures and 5 other FCS methods

Table S2. PCA loading coefficients for each MIPA measure

Table S3. Pairwise linear correlation coefficient between MIPA measures

Table S4. Comparative performance of MIPA vis-à-vis other FCS methods

Table S5. Pathway activity analysis of wound healing response

Table S6. Significant differentially expressed genes in wound healing response

Table S7. Gene set enrichment analysis of wound healing response: upregulated pathways

Table S8. Gene set enrichment analysis of wound healing response: downregulated pathways

Table S9. Gene set overlap analysis of wound healing response: upregulated pathways

Table S10. Gene set overlap analysis of wound healing response: downregulated pathways

Table S11. Pathways regulated in response to *Salmonella* infection in wild-type intestinal epithelial cells

Tables S12-S19. Pathway activity analysis of antifungal and antibacterial response in host immunity

Table S20. Gene set overlap analysis of key driver genes identified for TLR stimulus-specific response (see Supplementary Figure S11)

Figure S1

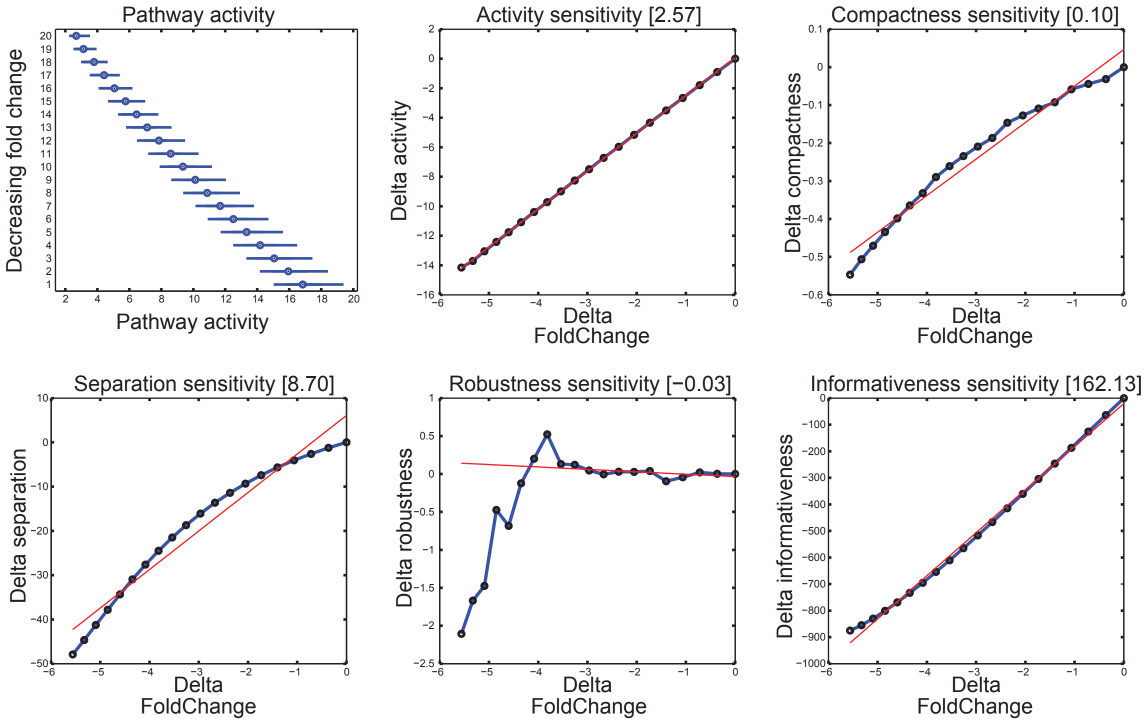
Pathway activity
Decreasing fold change
Pathway activity
Activity sensitivity [2.57]
Delta activity
Delta
FoldChange
Compactness sensitivity [0.10]
Delta compactness
Delta
FoldChange
Separation sensitivity [8.70]
Delta separation
Delta
FoldChange
Robustness sensitivity [−0.03]
Delta robustness
Delta
FoldChange
Informativeness sensitivity [162.13]
Delta informativeness
Delta
FoldChange

Figure S2

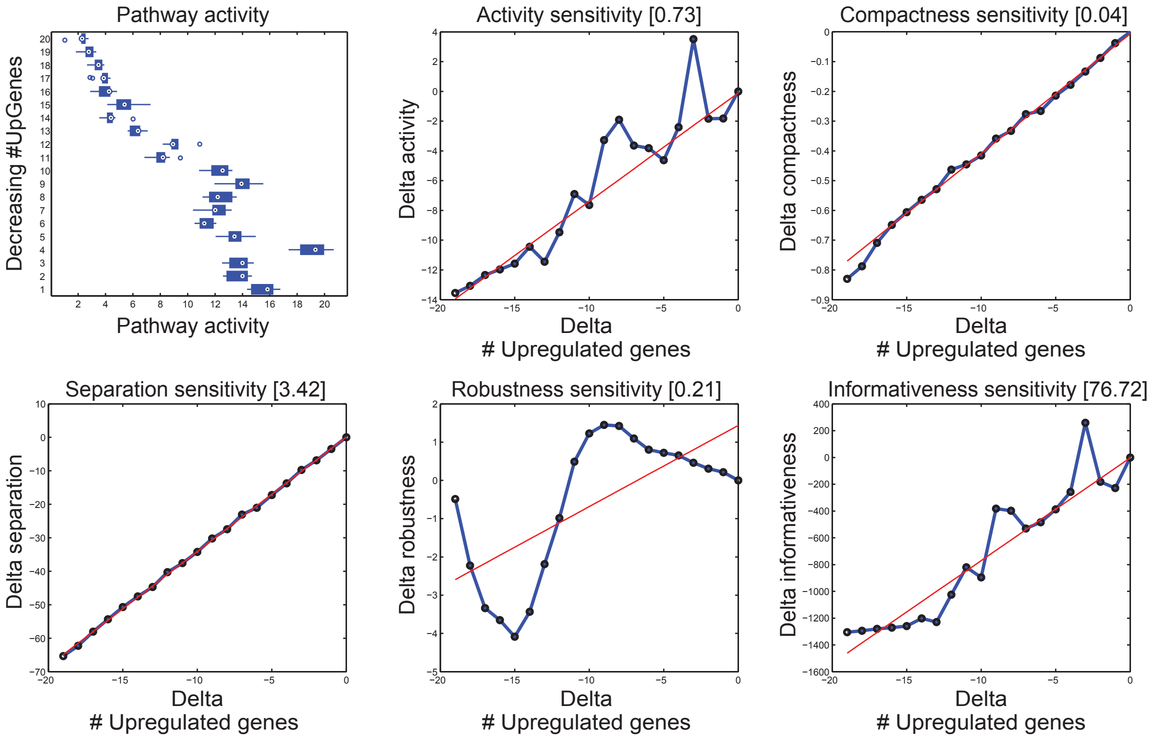
Pathway activity
Decreasing #UpGenes
Pathway activity
Activity sensitivity [0.73]
Delta activity
Delta
Upregulated genes
Compactness sensitivity [0.04]
Delta compactness
Delta
Upregulated genes
Separation sensitivity [3.42]
Delta separation
Delta
Upregulated genes
Robustness sensitivity [0.21]
Delta robustness
Delta
Upregulated genes
Informativeness sensitivity [76.72]
Delta informativeness
Delta
Upregulated genes

Figure S3

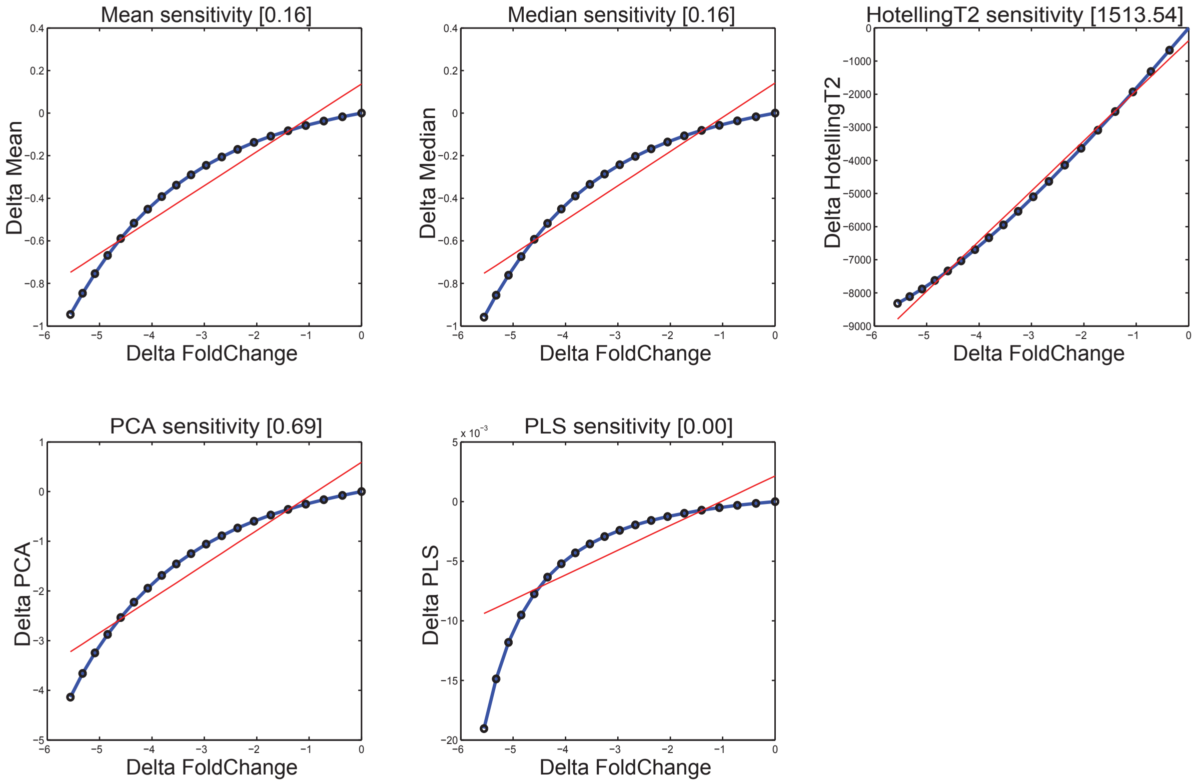
Mean sensitivity [0.16]
Delta Mean
Delta FoldChange
Median sensitivity [0.16]
Delta Median
Delta FoldChange
HotellingT2 sensitivity [1513.54]
Delta HotellingT2
Delta FoldChange
PCA sensitivity [0.69]
Delta PCA
Delta FoldChange
PLS sensitivity [0.00]
x 10^-3
Delta PLS
Delta FoldChange

Figure S4

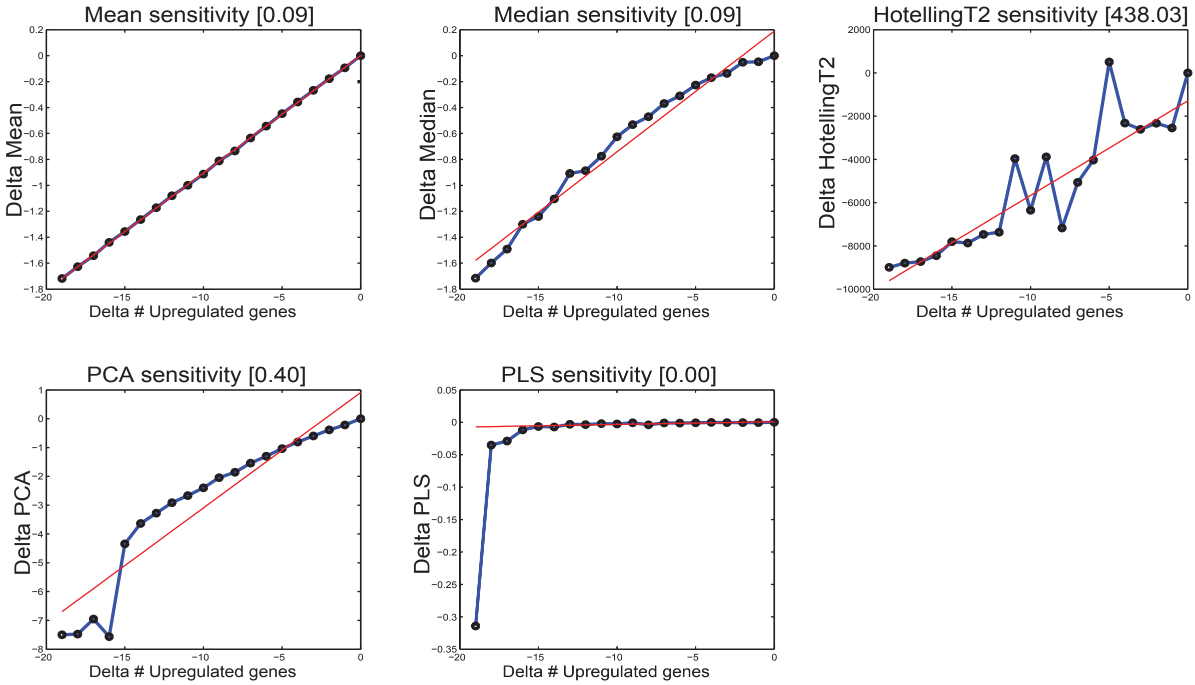
Mean sensitivity [0.09]
Delta Mean
Delta # Upregulated genes
Median sensitivity [0.09]
Delta Median
Delta # Upregulated genes
HotellingT2 sensitivity [438.03]
Delta HotellingT2
Delta # Upregulated genes
PCA sensitivity [0.40]
Delta PCA
Delta # Upregulated genes
PLS sensitivity [0.00]
Delta PLS
Delta # Upregulated genes

Figure S5

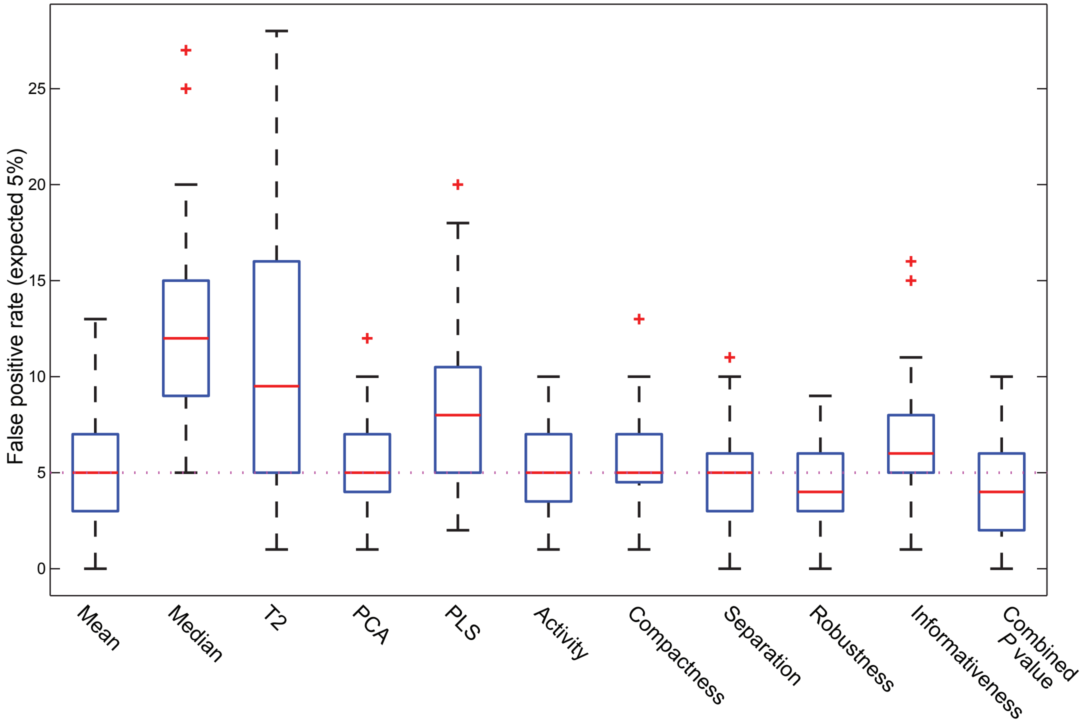

False positive rate (expected 5%)
0
5
10
15
20
25
Mean
Median
T2
PCA
PLS
Activity
Compactness
Separation
Robustness
Informativeness
Combined P value

Figure S6

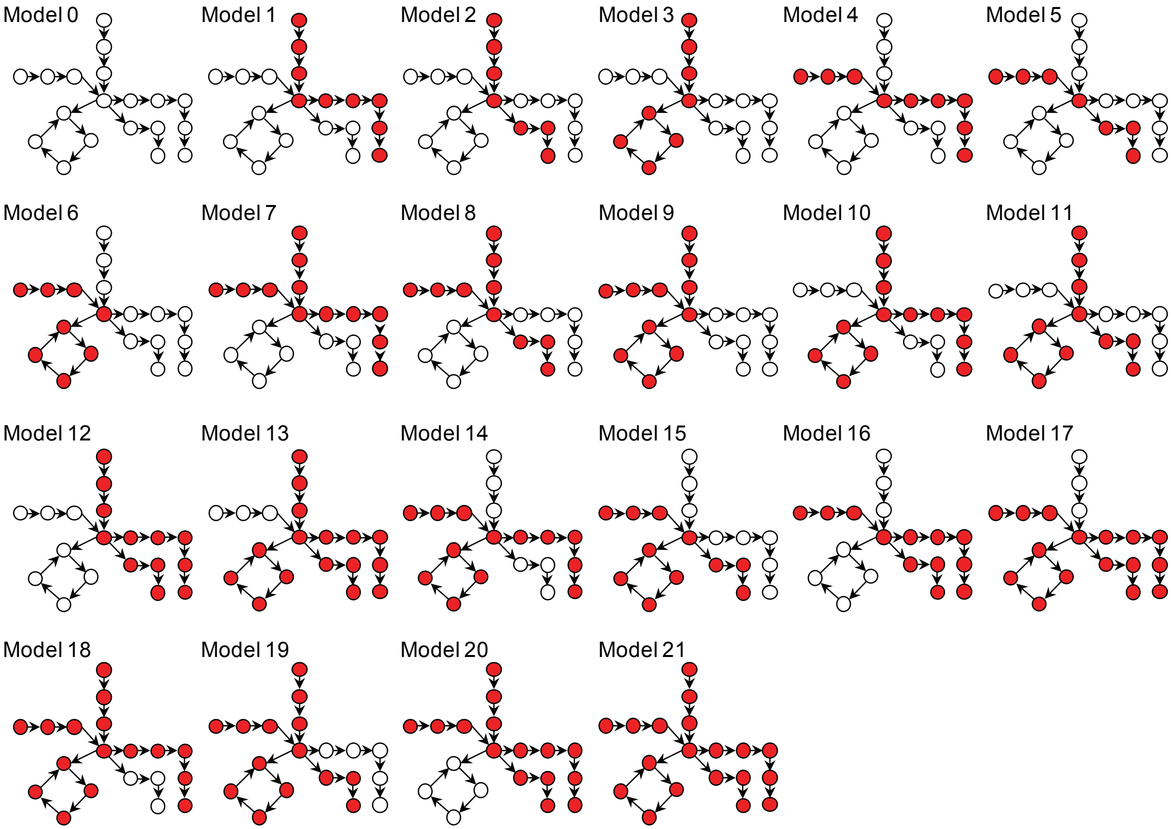

Model 0
Model 1
Model 2
Model 3
Model 4
Model 5
Model 6
Model 7
Model 8
Model 9
Model 10
Model 11
Model 12
Model 13
Model 14
Model 15
Model 16
Model 17
Model 18
Model 19
Model 20
Model 21

Figure S7

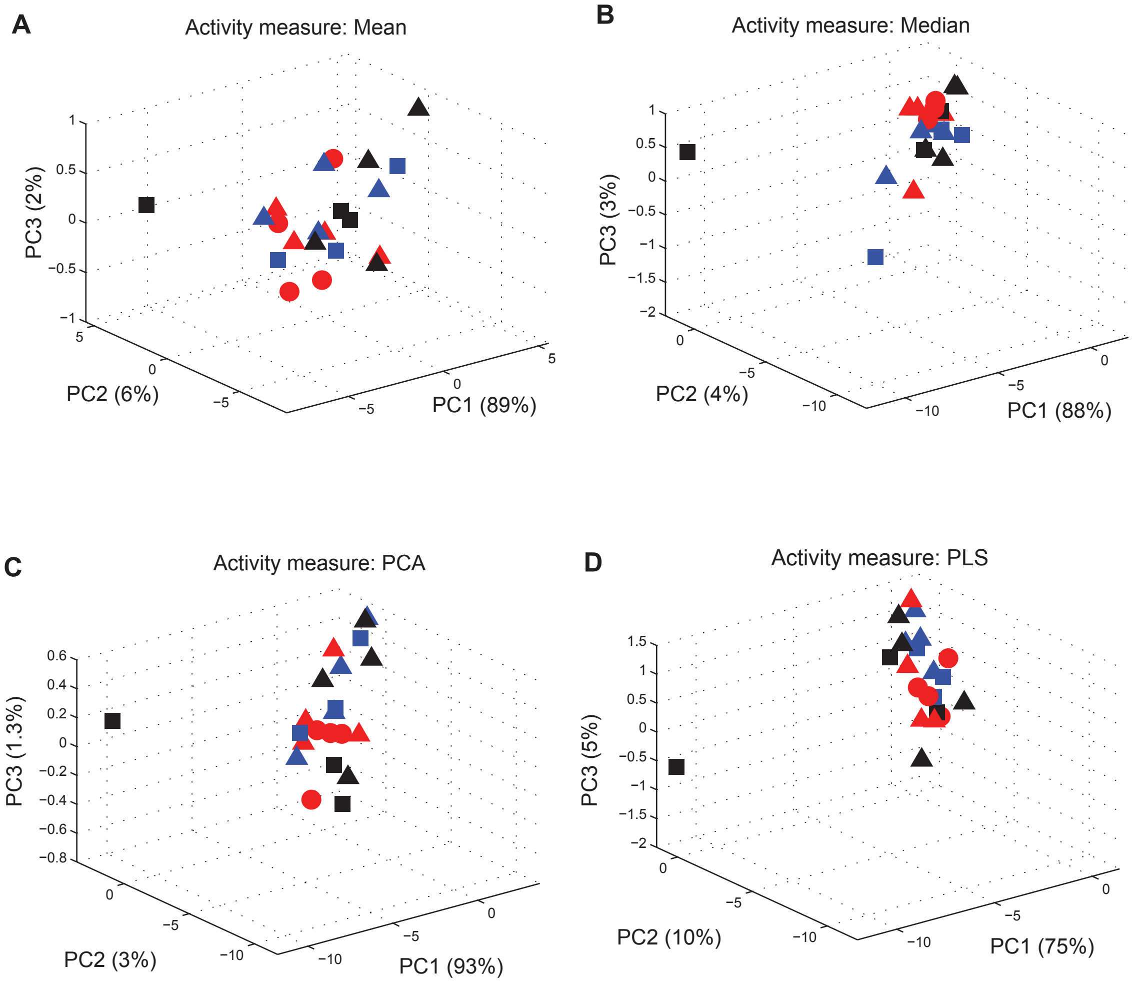
A
Activity measure: Mean
PC3 (2%)
PC2 (6%)
PC1 (89%)
B
Activity measure: Median
PC3 (3%)
PC2 (4%)
PC1 (88%)
C
Activity measure: PCA
PC3 (1.3%)
PC2 (3%)
PC1 (93%)
D
Activity measure: PLS
PC3 (5%)
PC2 (10%)
PC1 (75%)

Figure S8

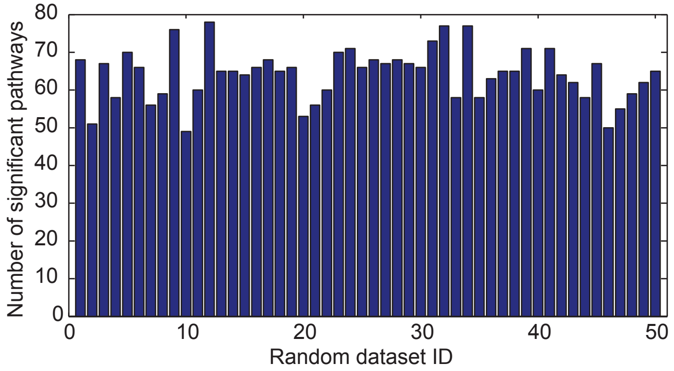
Number of significant pathways
0
10
20
30
40
50
60
70
80
0
10
20
30
40
50
Random dataset ID

Figure S9

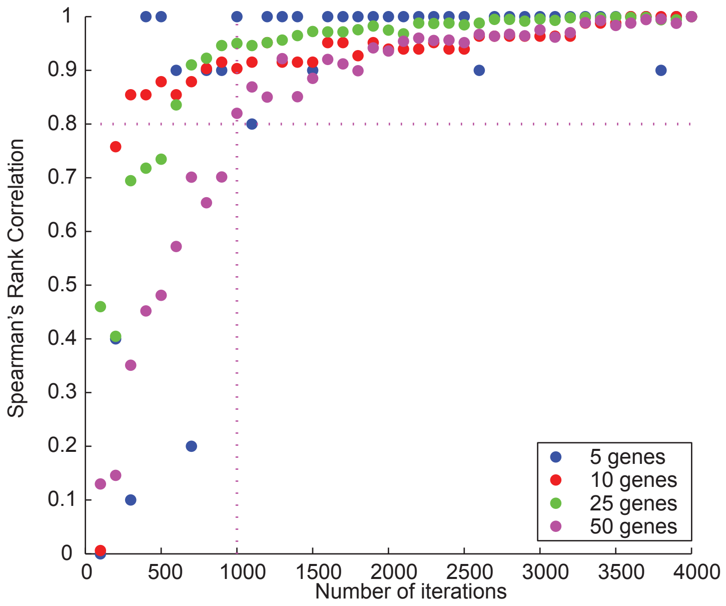

Spearman's Rank Correlation
0
0.1
0.2
0.3
0.4
0.5
0.6
0.7
0.8
0.9
1
0
500
1000
1500
2000
2500
3000
3500
4000
Number of iterations
5 genes
10 genes
25 genes
50 genes

Figure S10

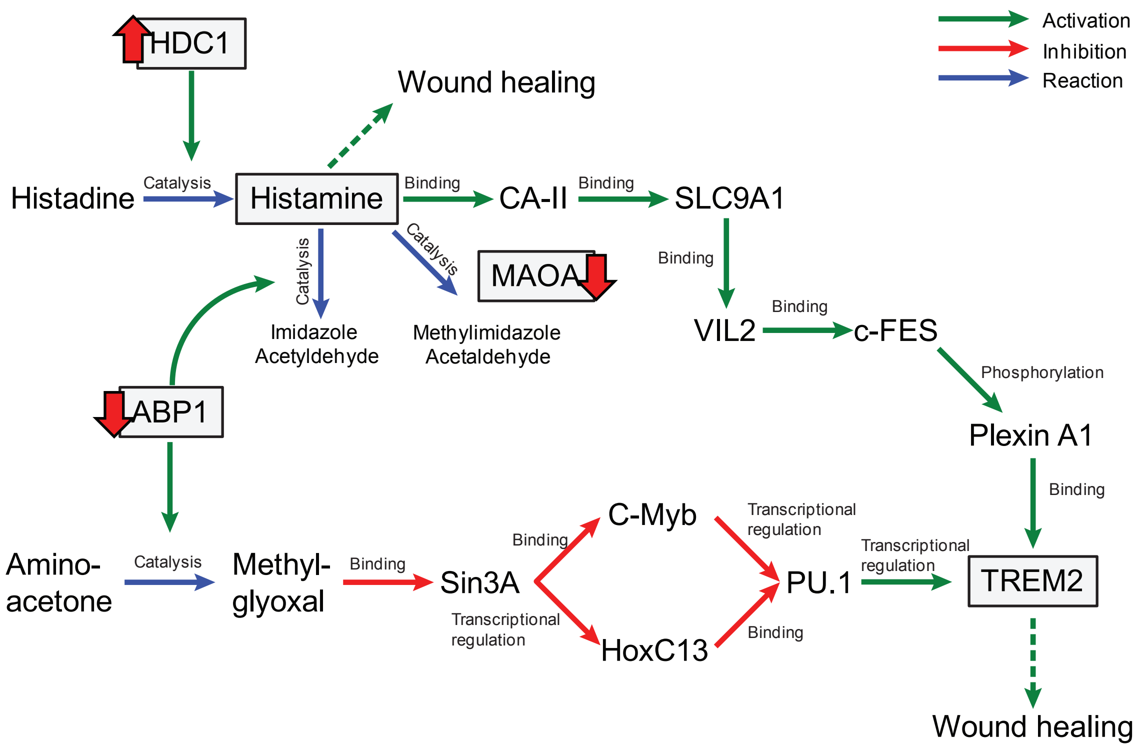

Activation
Inhibition
Reaction
HDC1
Wound healing
Histadine
Catalysis
Histamine
Binding
CA-II
Binding
SLC9A1
Catalysis
Catalysis
MAOA
Binding
Imidazole Acetyldehyde
Methylimidazole Acetaldehyde
VIL2
Binding
c-FES
Phosphorylation
ABP1
Plexin A1
Binding
C-Myb
Transcriptional regulation
Binding
Transcriptional regulation
Amino-acetone
Catalysis
Methyl-glyoxal
Binding
Sin3A
PU.1
TREM2
Transcriptional regulation
HoxC13
Binding
Wound healing

Figure S11

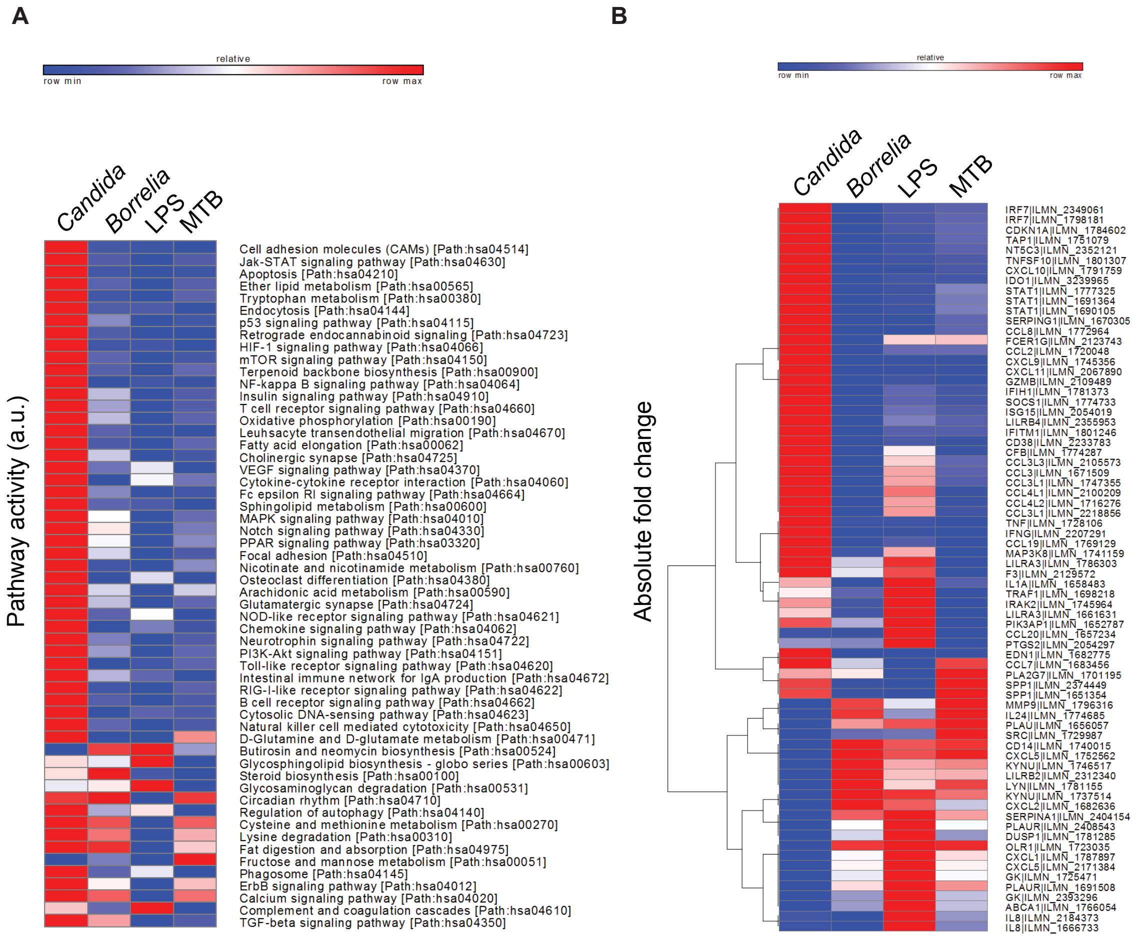
A
relative
row min
row max
Candida
Borrelia
LPS
MTB
Pathway activity (a.u.)
Cell adhesion molecules (CAMs) [Path:hsa04514]
Jak-STAT signaling pathway [Path:hsa04630]
Apoptosis [Path:hsa04210]
Ether lipid metabolism [Path:hsa00565]
Tryptophan metabolism [Path:hsa00380]
Endocytosis [Path:hsa04144]
p53 signaling pathway [Path:hsa04115]
Retrograde endocannabinoid signaling [Path:hsa04723]
HIF-1 signaling pathway [Path:hsa04066]
mTOR signaling pathway [Path:hsa04150]
Terpenoid backbone biosynthesis [Path:hsa00900]
NF-kappa B signaling pathway [Path:hsa04064]
Insulin signaling pathway [Path:hsa04910]
T cell receptor signaling pathway [Path:hsa04660]
Oxidative phosphorylation [Path:hsa00190]
Leuhsacyte transendothelial migration [Path:hsa04670]
Fatty acid elongation [Path:hsa00062]
Cholinergic synapse [Path:hsa04725]
VEGF signaling pathway [Path:hsa04370]
Cytokine-cytokine receptor interaction [Path:hsa04060]
Fc epsilon RI signaling pathway [Path:hsa04664]
Sphingolipid metabolism [Path:hsa00600]
MAPK signaling pathway [Path:hsa04010]
Notch signaling pathway [Path:hsa04330]
PPAR signaling pathway [Path:hsa03320]
Focal adhesion [Path:hsa04510]
Nicotinate and nicotinamide metabolism [Path:hsa00760]
Osteoclast differentiation [Path:hsa04380]
Arachidonic acid metabolism [Path:hsa00590]
Glutamatergic synapse [Path:hsa04724]
NOD-like receptor signaling pathway [Path:hsa04621]
Chemokine signaling pathway [Path:hsa04062]
Neurotrophin signaling pathway [Path:hsa04722]
PI3K-Akt signaling pathway [Path:hsa04151]
Toll-like receptor signaling pathway [Path:hsa04620]
Intestinal immune network for IgA production [Path:hsa04672]
RIG-I-like receptor signaling pathway [Path:hsa04622]
B cell receptor signaling pathway [Path:hsa04662]
Cytosolic DNA-sensing pathway [Path:hsa04623]
Natural killer cell mediated cytotoxicity [Path:hsa04650]
D-Glutamine and D-glutamate metabolism [Path:hsa00471]
Butirosin and neomycin biosynthesis [Path:hsa00524]
Glycosphingolipid biosynthesis - globo series [Path:hsa00603]
Steroid biosynthesis [Path:hsa00100]
Glycosaminoglycan degradation [Path:hsa00531]
Circadian rhythm [Path:hsa04710]
Regulation of autophagy [Path:hsa04140]
Cysteine and methionine metabolism [Path:hsa00270]
Lysine degradation [Path:hsa00310]
Fat digestion and absorption [Path:hsa04975]
Fructose and mannose metabolism [Path:hsa00051]
Phagosome [Path:hsa04145]
ErbB signaling pathway [Path:hsa04012]
Calcium signaling pathway [Path:hsa04020]
Complement and coagulation cascades [Path:hsa04610]
TGF-beta signaling pathway [Path:hsa04350]
B
relative
row min
row max
Candida
Borrelia
LPS
MTB
Absolute fold change
IRF7|ILMN_2349061
IRF7|ILMN_1798181
CDKN1A|ILMN_1784602
TAP1|ILMN_1751079
NT5C3|ILMN_2352121
TNFSF10|ILMN_1801307
CXCL10|ILMN_1791759
IDO1|ILMN_3239965
STAT1|ILMN_1777325
STAT1|ILMN_1691364
STAT1|ILMN_1690105
SERPING1|ILMN_1670305
CCL8|ILMN_1772964
FCER1G|ILMN_2123743
CCL2|ILMN_1720048
CXCL9|ILMN_1745356
CXCL11|ILMN_2067890
GZMB|ILMN_2109489
IFIH1|ILMN_1781373
SOCS1|ILMN_1774733
ISG15|ILMN_2054019
LILRB4|ILMN_2355953
IFITM1|ILMN_1801246
CD38|ILMN_2233783
CFB|ILMN_1774287
CCL3L3|ILMN_2105573
CCL3|ILMN_1671509
CCL3L1|ILMN_1747355
CCL4L1|ILMN_2100209
CCL4L2|ILMN_1716276
CCL3L1|ILMN_2218856
TNF|ILMN_1728106
IFNG|ILMN_2207291
CCL19|ILMN_1769129
MAP3K8|ILMN_1741159
LILRA3|ILMN_1786303
F3|ILMN_2129572
IL1A|ILMN_1658483
TRAF1|ILMN_1698218
IRAK2|ILMN_1745964
LILRA3|ILMN_1661631
PIK3AP1|ILMN_1652787
CCL20|ILMN_1657234
PTGS2|ILMN_2054297
EDN1|ILMN_1682775
CCL7|ILMN_1683456
PLA2G7|ILMN_1701195
SPP1|ILMN_2374449
SPP1|ILMN_1651354
MMP9|ILMN_1796316
IL24|ILMN_1774685
PLAU|ILMN_1656057
SRC|ILMN_1729987
CD14|ILMN_1740015
CXCL5|ILMN_1752562
KYNU|ILMN_1746517
LILRB2|ILMN_2312340
LYN|ILMN_1781155
KYNU|ILMN_1737514
CXCL2|ILMN_1682636
SERPINA1|ILMN_2404154
PLAUR|ILMN_2408543
DUSP1|ILMN_1781285
OLR1|ILMN_1723035
CXCL1|ILMN_1787897
CXCL5|ILMN_2171384
GK|ILMN_1725471
PLAUR|ILMN_1691508
GK|ILMN_2393296
ABCA1|ILMN_1766054
IL8|ILMN_2184373
IL8|ILMN_1666733

Figure S12

A

Infliximab response pathways

1. Bile secretion [Path:hsa04976]
2. Complement and coagulation cascades [Path:hsa04610]
3. Endocrine and other factor-regulated calcium reabsorption [Path:hsa04961]
4. Fatty acid metabolism [Path:hsa00071]
5. Fc epsilon RI signaling pathway [Path:hsa04664]
6. GABAergic synapse [Path:hsa04727]
7. Gastric acid secretion [Path:hsa04971]
8. Glutathione metabolism [Path:hsa00480]
9. Glycolysis / Gluconeogenesis [Path:hsa00010]
10. Glyoxylate and dicarboxylate metabolism [Path:hsa00630]
11. Histidine metabolism [Path:hsa00340]
12. Intestinal immune network for IgA production [Path:hsa04672]
13. Metabolism of xenobiotics by cytochrome P450 [Path:hsa00980]
14. NF-kappa B signaling pathway [Path:hsa04064]
15. PI3K-Akt signaling pathway [Path:hsa04151]
16. Primary bile acid biosynthesis [Path:hsa00120]
17. Steroid hormone biosynthesis [Path:hsa00140]
18. Synthesis and degradation of ketone bodies [Path:hsa00072]

B

**HMGCS2**	3-hydroxy-3-methylglutaryl-CoA synthase 2 (mitochondrial)	Synthesis and degradation of ketone bodies [Path:hsa00072]
**AQP8**	aquaporin 8	Bile secretion [Path:hsa04976]
**ABCB1**	ATP-binding cassette, sub-family B (MDR/TAP), member 1	Bile secretion [Path:hsa04976]
**ABCB1**	ATP-binding cassette, sub-family B (MDR/TAP), member 1	Bile secretion [Path:hsa04976]
**UGT2A3**	UDP glucuronosyltransferase 2 family, polypeptide A3	Steroid hormone biosynthesis [Path:hsa00140]
**KLK1**	kallikrein 1	Endocrine and other factor-regulated calcium reabsorption [Path:hsa04961]
**SLC4A4**	solute carrier family 4, sodium bicarbonate cotransporter, member 4	Bile secretion [Path:hsa04976]
**HMGCS2**	3-hydroxy-3-methylglutaryl-CoA synthase 2 (mitochondrial)	Synthesis and degradation of ketone bodies [Path:hsa00072]
**CFTR**	cystic fibrosis transmembrane conductance regulator (ATP-binding cassette sub-family C, member 7)	Bile secretion [Path:hsa04976]; Gastric acid secretion [Path:hsa04971]
**PROS1**	protein S (alpha)	Complement and coagulation cascades [Path:hsa04610]
**CFTR**	cystic fibrosis transmembrane conductance regulator (ATP-binding cassette sub-family C, member 7)	Bile secretion [Path:hsa04976]
**AMACR**	alpha-methylacyl-CoA racemase	Primary bile acid biosynthesis [Path:hsa00120]
**SLC4A4**	solute carrier family 4, sodium bicarbonate cotransporter, member 4	Bile secretion [Path:hsa04976]
**BDH1**	3-hydroxybutyrate dehydrogenase, type 1	Synthesis and degradation of ketone bodies [Path:hsa00072]
